# Comparison of reverse hybridization and *ompA* sequencing methods applied on *Chlamydia trachomatis* strains from Tunisia

**DOI:** 10.1002/mbo3.549

**Published:** 2017-12-28

**Authors:** Houda Gharsallah, Olfa Frikha‐Gargouri, Reinier J. Bom, Adnene Hammami, Sylvia M. Bruisten

**Affiliations:** ^1^ Department of Microbiology and research laboratory “Microorganismes et Pathologies Humaines” Habib Bourguiba university hospital Medical School of Sfax University of Sfax Sfax Tunisia; ^2^ Biopesticides Laboratory Centre of Biotechnology of Sfax University of Sfax Sfax Tunisia; ^3^ Public Health Laboratory The Netherlands Condomerie Cluster Infectious Diseases Public Health Service Amsterdam Amsterdam The Netherlands; ^4^ Public Health Laboratory Public Health Service of Amsterdam (GGD Amsterdam) Amsterdam the Netherlands

**Keywords:** *Chlamydia trachomatis*, comparison, genotyping, *ompA* sequencing, reverse hybridization method

## Abstract

Two techniques based on *ompA* amplification of *Chlamydia trachomatis* were compared, being reverse hybridization (RHM) and *ompA* sequencing (OSA), to investigate the concordance between them and to study the epidemiological relevance of each method. In addition, phylogenetic analysis was performed on the *ompA* sequences. One hundred and seven *C. trachomatis* positive samples from Tunisian patients and female sex workers were analyzed using both the RHM and *ompA* sequencing. The overall genovar distribution obtained with both techniques was very similar. The RHM identified nine genovars, being B, D, E, F, G, H, I, J and K, where B, I, J, and K were only found in mixed infections versus 7 types for the OSA being D, E, F, G, H, I, and K. The agreement between both typing techniques was 87.8%. Both methods showed that genovar E was the most predominant type. In 24.3% of the analyzed samples, mixed infections were detected. In 96.1% of these, the genovar identified by OSA was also detected using the RHM. O*mpA* sequencing allowed determination of six genovar types that could not be typed using RHM. The analyses of *ompA* nucleotide variation in the 107 clinical specimens detected *ompA* genovar variants with distinct *ompA* mutational patterns for types D2, G1, G2, and H1. In conclusion, RHM and OSA showed a high agreement in *C. trachomatis* genotyping results with each having their specific benefits.

## 1 INTRODUCTION

*Chamydia trachomatis* is the most frequent cause of curable sexually transmitted infections worldwide. The *ompA* is one of the most variable genes in the *Chlamydiae* genomes. It contains four highly polymorphic variable domains (VD1–4) separated by five constant domains (CD1–5). The most variable nucleotide sequences are found in the VD1 and VD2 regions, thus being the most discriminatory regions. These sequences were exploited by all typing techniques targeting a fragment of this gene (Spaargaren, Fennema, Morré, de Vries, & Coutinho, 2005).

Using the *ompA* gene target which encodes the major outer membrane protein (MOMP), genovars A to C are predominantly associated with trachoma and are rarely detected in urogenital diseases. Genovars D to K are associated with urogenital diseases, and genovars L1 to L3 are associated with lymphogranuloma venereum (LGV) (Andersson et al., 2016; Yuan, Zang, Watkins, & Caldwell, 1989).

Characterization of *C. trachomatis* genovars is based on techniques that can be helpful to clarify the spread of strains in epidemiological studies. These techniques are also useful in clinical studies by investigation of person‐to‐person transmission, assessing the relationship between genovars and studying responses to treatment between them. Moreover, characterization of *C. trachomatis* strains can provide important knowledge for improved control measures.

The first developed molecular method for *C. trachomatis* genotyping consisted of the amplification of the *ompA* gene followed by restriction fragment length polymorphism (PCR‐RFLP). This technique requires no specialized instruments and hence it was widely applied in epidemiological studies of *C. trachomatis* genotyping (Foschi et al., 2016; Petrovay, Németh, Balázs, & Balla, 2015; Rodriguez et al., 1991; Taheri Beni et al., 2012). Even though the PCR‐RFLP has its own limitations and many alternative techniques, such as sequencing analysis, are available, it is still used nowadays in many countries and laboratories. Next, *ompA* sequencing was developed. This is still the method of choice for strain differentiation because it is more discriminating than PCR‐RFLP and allowed the discovery of new genovariants (Brasiliense, Borges, & Ferreira, 2016; Lan, Ossewaarde, Walboomers, Meijer, & van den Brule, 1994; Lysén et al., 2004; Martínez, Ovalle, Camponovo, & Vidal, 2015; Morré, Ouburg, van Agtmael, & de Vries, 2008). However, sequencing techniques were not really adequate to detect mixed genovar infections. In contrast, hybridization methods targeting the variable regions of the *ompA* gene are very well suited to detect co‐infections or mixed infections, and several hybridization based assays are available nowadays (Gharsallah, Frikha‐Gargouri, Besbes, et al., 2012a; Quint et al., 2007; Ruettger et al., 2011; Xiong, Kong, Zhou, & Gilbert, 2006; Zheng et al., 2007). These hybridization methods are suitable for epidemiological studies and are preferred because of their low costs and low technology requirement.

In this study, 107 Tunisian samples were analyzed for *C. trachomatis* genovars detection using the reverse hybridization method (RHM) and *ompA* gene sequence analysis (OSA). We aimed to characterize and compare the differences between the two techniques and to assess the genetic variants of part of the *ompA* gene in a set of samples derived from Tunisian patients and female sex workers.

## 2 MATERIAL AND METHODS

### 2.1 Clinical *Chlamydia trachomatis* samples

A total of 107 *C. trachomatis*‐positive samples from the microbiology laboratory, Habib Bourguiba, University Hospital of Sfax, were included in this study. Of the 107 samples, 85 were from clinical patients (CP) attending the STD clinics and 22 were from female sex workers (FSW) (Gharsallah, Frikha‐Gargouri, Besbes, et al., 2012b). Urethral and endocervical samples were analyzed through the Cobas Amplicor testing for the detection of *C. trachomatis* infection. Samples in 2SP transport medium were kept at −80°C until genotyping.

### 2.2 Extraction of genomic DNA

Genomic DNA was extracted from 200 μl of urethral and endocervical swabs using the QIAmp DNA Minikit (QIAGEN GmbH, Hilden, Germany) according to the manufacturer's instructions for all stored and available *C. trachomatis* positive samples. The DNA was then eluted in 50 μl of the elution buffer and stored at −20°C. The extracted DNA was tested for the human ß‐globin gene to check for PCR inhibitors in the samples. Primers ß‐GPCO (5′‐ACACAACTGTGTTCACTAGC‐ 3′), and ß‐GPCPO (5′GAAACCCAAGAGTCTTCTCT‐ 3′), were used to amplify a 209 bp fragment of the human ß‐globin gene to assess extraction.

### 2.3 Reverse hybridization method for genotyping

A semi‐nested PCR was used for the amplification of the region spanning the VD1 and VD2. The reverse hybridization method, developed in our laboratory, was used for the detection of specific *C*. *trachomatis* genovars (Gharsallah, Frikha‐Gargouri, Besbes, et al., 2012a, Gharsallah, Frikha‐Gargouri, Sellami, et al., 2012b). Briefly, the nylon membrane was spotted with probes hybridizing specifically to genovars A to L3 at specific positions. The reverse hybridization probes were then attached to the membrane by baking at 120°C for 30 min in an oven (Memmert, Germany). The membranes were pre‐hybridized for 30 min at 37°C and then hybridization took place overnight at 37°C with shaking. The biotinylated duplex DNA‐probe was revealed after incubation with the alkaline phosphatase enzyme for one hour at 37°C and then with its substrate, Western Blue stabilized substrate (Promega, Madison WI, USA); for 30 min at room temperature and in the dark. Washing was performed between each of the incubation steps.

### 2.4 *ompA* sequencing

The DNA was amplified by a nested PCR targeting the *ompA* VD1‐VD2 using a C1000 PCR machine (Bio‐Rad, Veenendaal, The Netherlands). The labeled DNA was purified using ABI BigDye XTerminator kit (Applied Biosystems) and analyzed in an ABI 3130 genetic analyzer (Applied Biosystems). Selected primers for the amplification and sequencing of the *ompA* VD1‐VD2 fragment was previously published for MLST studies (Bom et al., 2011).

The obtained sequences were compared to those of *C. trachomatis* reference strains using MEGA 7.0. Each obtained sequence was aligned with an analogous sequence from reference strains. The *C. trachomatis ompA* sequences accessed in GenBank are for genovar A, accession no. M33635; genovar B: AF063208; genovar C: AF352789; genovar D: AY535082; genovar E: AY535111; genovar F: AY464145; genovar G: AF063199; genovar H: X16007; genovar I: AF063200; genovar J: AF086856; genovar K: AF265239; genovar L1: M36533; genovar L2: M14738 and genovar L3: X55700.

### 2.5 Ethical statement

The study has been approved by the institutional ethics committee of Habib Bourguiba university hospital. All samples used in this study were re‐coded in order to anonymize patients’ information prior to analysis. Therefore no personal information was linked to these specimens and individuals could not be matched with their samples and their epidemiological and clinical data. Samples and epidemiological data were collected for diagnostic purposes under standard of care protocols for sexually transmitted infections in each location. Oral consent, as approved by our institutional Ethics Committee, was obtained.

### 2.6 Statistical analysis

Data were analyzed using SPSS version 19.0. Differences between the studied groups (CP, FSW, and genovars) were statistically compared by the Chi‐squared test or the Fisher's exact test when sample sizes were small *p *<* *.05 was considered as significant.

## 3 RESULTS

### 3.1 Genovar distribution determined by the reverse hybridization method

Using RHM, *C. trachomatis* samples showed nine genovar types B, D, E, F, G, H, I, J, and K of which B, I, J, and K were only found in mixed infections (Table S1). Genovar types A, C, and LGV were not detected in these samples. In 6/107 samples (5.6%), no genovar type could be determined using RHM. Single genovars were detected in 75/107 (70.1%) and mixed genovars in 26/107 (24.3%).

**Figure 1:**
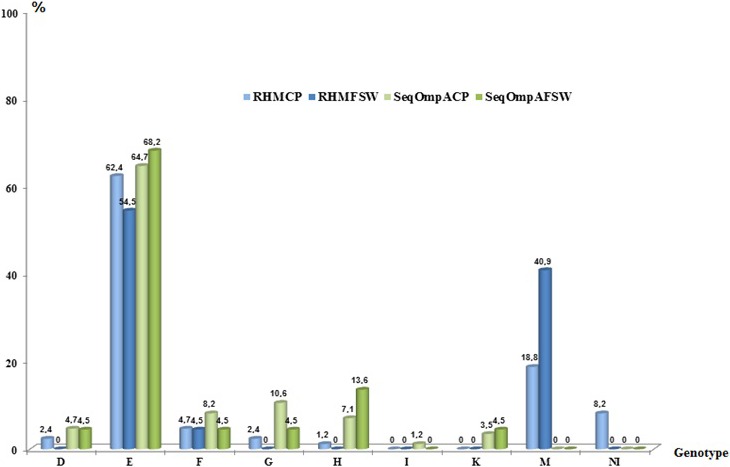
Distribution of the 107 *C. trachomatis* genovars of CP and FSW populations by both the Reverse Hybridization Method (RHM) and *ompA* sequencing. M: mixed infections; NI: Not Identified genovars by RHM; RHM‐CP: Reverse Hybridization Method Clinical Patients; RHM‐FSW: Reverse Hybridization Method Female Sex Workers; Seq*ompA‐* CP: Sequencing o*mpA* Clinical Patients; Seq *ompA‐* FSW: Sequencing *ompA* Female Sex Workers; CP, clinical patients; FSW, female sex workers

Figure 1 shows the typing results separately for the 85 CP attending the STD clinics and the 22 FSW. For the CP group, using RHM no type could be determined for 6 samples. Of the 79 typed CP samples, single infections were observed in 62/79 (78.5%) of cases and mixed infections in 17/79 (21.5%). Double infections (two genovars) occurred most often in the CP group 14/79 (17.7%), with 4/79 (5.1%) for the combinations E + G and E + H.

Comparing the prevalence of *C. trachomatis* genovars between the CP and the FSW groups with the RHM technique, genovar E was by far the most prevalent type (including samples with mixed infections), reaching 68/85 (80.0%) and 20/22 (90.9%), respectively (Table S1). The E + F combination was most frequently detected in 3/22 (13.6%) of the FSW. Mixed infections with four genovars were detected in 2/22 (9.1%) of FSW cases.

### 3.2 Genovar distribution determined by *ompA* sequencing

Considering *ompA* sequencing, the *C. trachomatis* prevalence for CP and FSW was distributed differently (Table S1, Figure 1). In the CP group, genovar E was the most prevalent one (64.7%) followed by G (10.6%), F (8.2%), H (7.1%), D (4.7%), K (3.5%), and I (1.2%). For the FSW group, genovar E was also the most prevalent type (68.2%), followed by H (13.6%) and D, F, G, K (4.5% for each genovar). A difference in the prevalence of genovar H was observed between the FSW and women from the CP group, but that did not reach statistical significance (*p* > .05). Genovars F, H, and K were detected only in men in the CP group, but were also found in FSW. Genovar I was not detected in the FSW group.

### 3.3 Comparison of genovar distribution in Tunisian patients determined by the reverse hybridization method and *ompA* sequencing

All genovars that were detected by the RHM method were also identified using *ompA* sequencing, except genovars B and J which were identified only in mixed infections by RHM. Six cases yielding no identified genovars by the RHM were resolved by OSA and four of these were genovar E.

There were 75 cases with single infections by both RHM and OSA and 69 of these (92.0%) showed concordant genovar types (Table 1). In 6 out of the 64 genovar E cases identified by RHM, a different genovar was identified by sequencing, namely genovar D (*n* = 1), G (*n* = 3), I (*n* = 1) and K (*n* = 1) (Table 1).

**Table 1:** Comparison of genovars of *Chlamydia trachomatis* as detected by reverse hybridization method and *ompA* sequencing in 107 Tunisian samples

	Genovars identified by *ompA* sequencing							Total 107
	D	E	F	G	H	I	K	
*Genovars identified by reverse hybridization method*								
Single infection								
D	**2**	0	0	0	0	0	0	2
E	1	**58**	0	3	0	1	1	64
F	0	0	**5**	0	0	0	0	5
G	0	0	0	**3**	0	0	0	3
H	0	0	0	0	**1**	0	0	1
Mixed infection								
B + E	0	**1**	0	0	0	0	0	1
D + E	**1**	0	0	0	0	0	0	1
D + F	0	0	**1**	0	0	0	0	1
E + F	0	**3**	**1**	0	0	0	0	4
E + F + J + K	0	0	0	0	0	0	**1**	1
E + G	0	**1**	0	**4**	0	0	0	5
E + G + H + K	0	0	0	0	**1**	0	0	1
E + H	0	**2**	0	0	**3**	0	0	5
E + H + K	0	**1**	0	0	**2**	0	0	3
E + I	0	0	0	0	1	0	0	1
E + K	0	**1**	0	0	0	0	**1**	2
H + K	0	0	0	0	**1**	0	0	1
Not identified genovars	1	4	0	0	0	0	1	6

Cesults are marked in bold.

**Figure 2:**
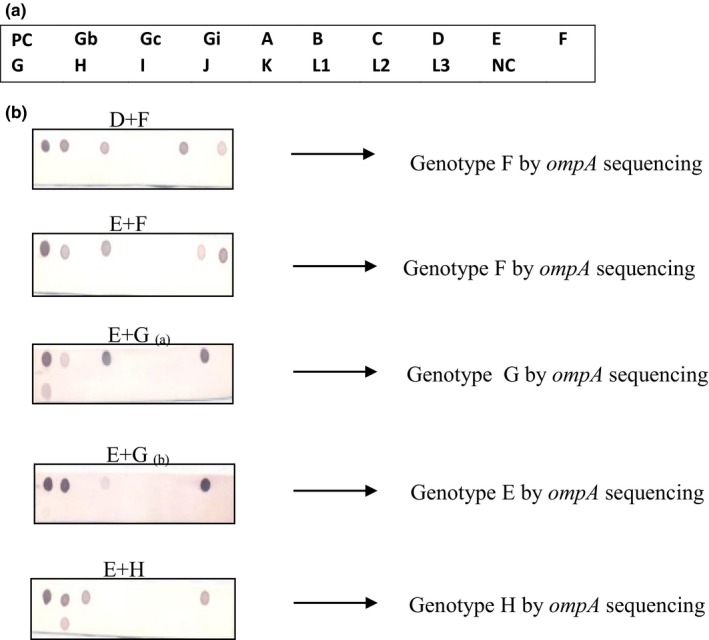
(a) The layout of the probes on the membrane: probes (A, B, C, D, E, F, G, H, I, J, K, L1, L2, and L3) and to the 3 group probes: the B group (Gb) representing genovars B, D, E, L1 and L2, the C group (Gc) representing genovars A, C, H, I, J, and L3 and the intermediate group (Gi) representing genovars F and G. Positive (PC) and Negative (NC) Controls. (b) Typing results of Reverse Hybridization Method (RHM) and the corresponding genotype identified by *ompA* sequencing

In 25/26 (96.1%) cases of mixed infections with RHM, the genovar detected by OSA was also present (Table 1). Only one mixed infection (E + I), typed by RHM, was identified as genovar H by OSA. In 9 of the 25 cases (36.0%) in which genovar E was present in mixed infections the presence of genovar E was confirmed by OSA. Figure 2 shows the results of some hybridization membranes and their corresponding OSA results. A closer look at the membranes showing mixed infections indicated that in most of them, the genovar and its corresponding group showing the highest intensities were those identified by OSA (see (E + F), (E + G_(b)_ in Figure 2). However, in few cases, the genovar corresponding to the faint spot was the one that was identified by OSA as observed in samples with genovars (E + G_(a)_) (Figure 2).

### 3.4 Sequence analysis of *ompA Chlamydia trachomatis* samples and genetic variants

Sequence analysis of the 590‐bp fragment of the *ompA* gene comprising VD1 and VD2 was performed. In general, the *ompA* amplified sequences of the 107 clinical specimens did not show many changes compared to those of reference strains (Table 2). In fact, genovar E, F, I, and K showed no genetic variation at all. There were nine distinct *ompA* mutations in genovars D1, D2, E, F, G1, G2, H1, I, and K among the 107 clinical specimens. From the 11 polymorphic sites; most mutations (9/11) occurred in CD1, one in CD2 and one in VD2 region of the *ompA* gene. The highest number of mutations was observed in genovar D variant. Among the D2 variant, 8‐point mutations were seen compared to the D reference strain AY535082 in which one point mutation leads to an amino‐acid deletion (Table 2).

**Table 2:** Mutations found in *ompA* regions VD1‐ VD2 of 107 *Chlamydia trachomatis* samples in comparison to the GenBank reference strains

Genotype	Strains with mutation/total	No of nucleotide mutations	Nucleotide mutation	Mutation position	Domain in *ompA* gene	Amino acid change
D1	2/5	0	‐	‐	‐	‐
D2	3/5	9	T→C	248	CD1	V→A
			A→G	270		W→L
			A→G	300		M→T
			G→T	302		
			T→C	311		W→* (Deletion)
			G→A	339		Q→L
			A→T	344		S→F
			C→T	362		T→R
			C→G	365		
G1	9/10	1	A→G	487	CD2	G→S
G2	1/10	1	A→T	228	CD1	Silent
H1	9/9	1	A→G	440	VD2	N→S

## 4 DISCUSSION

*Chlamydia trachomatis ompA* sequencing analysis has been widely used for epidemiological studies because of its high resolution and discriminative power to detect polymorphism in the *ompA* gene. Although the problem of resolution for *C. trachomatis* typing is resolved by sequencing, the differentiation of mixed genovar infections is not possible. This issue could be resolved using hybridization genotyping methods. These emerged as an improvement in detecting mixed infections and have been used in various epidemiological studies. RHM typing is easy to perform and does not require expensive machines.

A total of 107 *C. trachomatis*‐positive uro‐genital specimens from Tunisian clinical patients and female sex workers were examined and successfully genotyped using the RHM and OSA. From the four variable domains of the *ompA* gene, the region spanning VD1 and VD2 was used in both techniques to determine the genovar. This region was demonstrated to be sufficiently polymorphic to differentiate between all *C. trachomatis* genovars (Nunes et al., 2009; Yuan et al., 1989).

Comparison of genovar distributions in Tunisian patients determined by the RHM and OSA showed that the overall distribution obtained with both techniques was similar with an agreement of 86% (92/107). This percentage was comparable to that reported when comparing results of PCR‐RFLP and DNA microarray which was 90% (Gallo Vaulet et al., 2016). Discrepancies of genotyping methods were also reported in the literature in which evidence of mixed infections or recombination events were suggested for discrepant samples (Joseph et al., 2014). Discordant results in our study were found almost exclusively in cases of mixed infections detected by RHM. This latter allows multiple genovars to be detected simultaneously on one nylon membrane by yielding different spot intensities. A technical explanation for the discrepancies would be a difference in the sensitivities of DNA amplification and hybridization of the probes of RHM in case of mixed genovars and/or the presence of mutations in the primer/probe regions used for PCR sequencing and for RHM. Also, low DNA quantities in the sample may influence detection of certain types in case of mixed infections. The spot of the genovar presenting the highest signal intensity with RHM was in most cases the one that was detected by *ompA* sequencing. Sanger sequencing is known to underestimate the number of genovars present in a given clinical sample because the most abundant genovar will be favored during PCR amplification at the expense of the others that may remain undetectable (Pedersen, Herrmann, & Møller, 2009). A possible limitation of RHM is that cross‐hybridization may occur, which would also be visible as faint spots and which lower the specificity of the assay. The RHM used in this study was previously validated before use, and its specificity was very high (Gharsallah, Frikha‐Gargouri, Besbes, et al., 2012a).

Mixed infections may result from two separate episodes of infection and the lack of immunological cross protection between genovars (Hsu et al., 2006). Reverse hybridization methods have proved to be of use in many studies to detect multiple infections (Gharsallah, Frikha‐Gargouri, Besbes et al., 2012a; Li et al., 2015; Quint et al., 2007; Xiong et al., 2006; Zheng et al., 2007). High frequencies of mixed infections were reported in studies using hybridization methods. In fact, Gallo Vaulet et al. (2016) reported that the detection of mixed‐genovar infections in South American samples was significantly higher using a microarray assay (8.4% of cases) compared to PCR‐RFLP (0.5%). The application of PCR‐reverse line blot hybridization assays by Molano, Meijer, Morré, Pol, and van den Brule (2004) showed that 9% of specimens from women in Colombia contained more than one *C. trachomatis* genovar. The application of the same technique by Li et al. (2015) showed a high frequency of *C. trachomatis* mixed infections (39.4%). Another study from China mentioned that the multiplex microsphere suspension array assay used for *C. trachomatis* genotyping detected in 18.3% of the samples multiple genotypes (Zhang et al., 2012).

*Chlamydia trachomatis* OSA is a very accurate method to determine sequence variation and it has been classified for decades as the standard for genotyping (Pedersen et al., 2009; De Vries, van der Loeff, & Bruisten, 2015). Indeed, in our study in 6 cases the genovar type could not be determined by RHM but this could be resolved by *ompA* sequencing analysis. The advantage of sequencing is that if the PCR primers fit, no a priori knowledge of the strain is needed, in contrast to the RHM, where probes need to be synthesized based on known sequences, which is a limitation of RHM. In our study, no insertion or deletion event was found in the *ompA* gene which is in agreement with Nunes et al. (2009) who reported that the *ompA* diversity is strictly a consequence of the occurrence of point mutations rather than recombination or indel events.

The variability in the *ompA* gene has been known for a long time (Frost, Deslandes, Gendron, Bourgaux‐Ramoisy, & Bourgaux, 1995; Sturm‐Ramirez et al., 2000). A recent study investigating 563 full genome sequences of *C. trachomatis* reported that the *ompA* gene experiences the highest rates of recombination in the genome (Hadfield et al., 2017). The presence of mixed infections could be explained by the possibility of recombination and fast diversification of *C. trachomatis* strains.

Genovar E was highly predominant in our study as reported previously in other studies world‐wide (Gharsallah et al., 2016; Hadfield et al., 2017; De Vries et al., 2015). The conservation of genovar E may be the result of either increased fitness or simply be a stochastic process, reflecting the behavior of the hosts (Hadfield et al., 2017; Nunes et al., 2009; Satoh, Ogawa, Saijo, & Ando, 2014; De Vries et al., 2015). An expansion of genovar E occurred in Sweden and Europe, due to failure to detect this strain by certain commercial assays. Also in Tunisia, this assay has been in use previously. In China, genovar E polymorphism was reported to be high reaching 12% as reported by Yang et al. (2010) reflecting different strains circulating in that area.

Nowadays, whole genome sequencing (WGS) offers the promise of precise acquisition of informative genetic information to even better distinguish *C. trachomatis* strains and use this in epidemiological studies. However, WGS is not practical for routine use since *C. trachomatis* grows intra‐cellularly, so it is difficult to obtain pure bacterial strains. Clinical samples are often collected in destructive buffers that do not allow enrichment of the bacteria with antibodies. Another obstacle for routine laboratories may be the need of a bioinformatics specialist to analyze the sequences. So, we argue that the RHM and OSA have their merits in their own right since RHM has the ability to detect dual or even multiple infections. In addition, it is simple to perform and does not require specialized expensive instruments. The OSA method is also relatively easy to perform, has the benefit to detect all genovar types (new genovars were determined in our study), still less expensive than WGS and giving quick information on genovar type to answer questions on re‐infections or treatment failures (Götz et al., 2013).

An important limitation of our study is that only 107 samples were available to be typed using the two techniques. A larger number of samples representing more different hosts is warranted to obtain more insight in the genetic variation in *C. trachomatis* strains in Tunisia, and surrounding North African countries.

In conclusion, a good agreement for genotyping results obtained by RHM and *ompA* sequencing was found. The use of RHM allowed the detection of mixed infections that were frequent, whereas the OSA permitted the detection of genetic variants.

## CONFLICT OF INTEREST

The authors declare that they have no competing interests.
