# Supplementary material for: Comparison of reverse hybridization and ompA sequencing methods applied on Chlamydia trachomatis strains from Tunisia
Source: Microbiologyopen. 2017 Dec 28;7(2):e00549. doi: 10.1002/mbo3.549 (PMC5911986; doi:10.1002/mbo3.549)
Supplement: Supplementary file 1 [file MBO3-7-na-s001.pdf]

Figure S1 *OmpA* sequences data

#CP_136
CCTTGCAAGCTCTGCCTGTGGGGAATCCTGCTGAACCAAGCCTTATGATCGACGGAATTCTGTGGGAAGGTTT
CGGTGGAGATCCTTGCGATCCTTGCACCACTTGGTGTGACGCTATCAGCATGCGTATGGGTTACTATGGAGAC
TTTGTTTTCGACCGTGTTTTGGAAACAGATGTGAATAAAGAATTCCACATGGGTGCCAAGCCTACAACTGATAC
AG--GCAATAGTGCA----
GCTCCATCCACTCTTACAGCAAGAGAGAATCCTGCTTACGGCCGACATATGCAGGATGCTGAGATGTTTACAA
ATGCCGCTTGCATGGCATTGAATATTTGGGATCGTTTTGATGTATTCTGTACATTAGGAGCCACCAGTGGATAT
CTTAAAGGAAACTCTGCTTCTTTCAATTTAGTTGGATTGTTTGGAGATAATGAAAATCAAAAAACGGTCAA---
AGCGGAGTCTGTACCAAATATGAGCTTTGATCAATCTGTTGTTGAGTTGTATACAGATACTACTTTTGCGTGGA
GCGTCGGCGCTCGCGCAGCTTTGTGGGAATGTGGATGTGCAACTTTAGGA

#FSW_45
CCTTGCAAGCTCTGCCTGTGGGGAATCCTGCTGAACCAAGCCTTATGATCGACGGAATTCTGTGGGAAGGTTT
CGGTGGAGATCCTTGCGATCCTTGCACCACTTGGTGTGACGCTATCAGCATGCGTATGGGTTACTATGGAGAC
TTTGTTTTCGACCGTGTTTTGGAAACAGATGTGAATAAAGAATTCCACATGGGTGCCAAGCCTACAACTGATAC
AG--GCAATAGTGCA----
GCTCCATCCACTCTTACAGCAAGAGAGAATCCTGCTTACGGCCGACATATGCAGGATGCTGAGATGTTTACAA
ATGCCGCTTGCATGGCATTGAATATTTGGGATCGTTTTGATGTATTCTGTACATTAGGAGCCACCAGTGGATAT
CTTAAAGGAAACTCTGCTTCTTTCAATTTAGTTGGATTGTTTGGAGATAATGAAAATCAAAAAACGGTCAA---
AGCGGAGTCTGTACCAAATATGAGCTTTGATCAATCTGTTGTTGAGTTGTATACAGATACTACTTTTGCGTGGA
GCGTCGGCGCTCGCGCAGCTTTGTGGGAATGTGGATGTGCAACTTTAGGA

#CP_141
CCTTGCAAGCTCTGCCTGTGGGGAATCCTGCTGAACCAAGCCTTATGATCGACGGAATTCTGTGGGAAGGTTT
CGGCGGAGATCCTTGCGATCCTTGCGCCACTTGGTGTGACGCTATCAGCATGCGTGTTGGTTACTACGGAGAC
TTTGTTTTCGACCGTGTTTTGAAAACTGATGTGAATAAAGAATTTCAGATGGGTGCCAAGCCTACAACTGATAC
AG--GCAATAGTGCA----
GCTCCATCCACTCTTACAGCAAGAGAGAATCCTGCTTACGGCCGACATATGCAGGATGCTGAGATGTTTACAA
ATGCCGCTTGCATGGCATTGAATATTTGGGATCGTTTTGATGTATTCTGTACATTAGGAGCCACCAGTGGATAT
CTTAAAGGAAACTCTGCTTCTTTCAATTTAGTTGGATTGTTTGGAGATAATGAAAATCAAAAAACGGTCAA---
AGCGGAGTCTGTACCAAATATGAGCTTTGATCAATCTGTTGTTGAGTTGTATACAGATACTACTTTTGCGTGGA
GCGTCGGCGCTCGCGCAGCTTTGTGGGAATGTGGATGTGCAACTTTAGGA

#CP_377
CCTTGCAAGCTCTGCCTGTGGGGAATCCTGCTGAACCAAGCCTTATGATCGACGGAATTCTGTGGGAAGGTTT
CGGCGGAGATCCTTGCGATCCTTGCGCCACTTGGTGTGACGCTATCAGCATGCGTGTTGGTTACTACGGAGAC
TTTGTTTTCGACCGTGTTTTGAAAACTGATGTGAATAAAGAATTTCAGATGGGTGCCAAGCCTACAACTGATAC
AG--GCAATAGTGCA----
GCTCCATCCACTCTTACAGCAAGAGAGAATCCTGCTTACGGCCGACATATGCAGGATGCTGAGATGTTTACAA
ATGCCGCTTGCATGGCATTGAATATTTGGGATCGTTTTGATGTATTCTGTACATTAGGAGCCACCAGTGGATAT
CTTAAAGGAAACTCTGCTTCTTTCAATTTAGTTGGATTGTTTGGAGATAATGAAAATCAAAAAACGGTCAA---
AGCGGAGTCTGTACCAAATATGAGCTTTGATCAATCTGTTGTTGAGTTGTATACAGATACTACTTTTGCGTGGA
GCGTCGGCGCTCGCGCAGCTTTGTGGGAATGTGGATGTGCAACTTTAGGA

#CP_380
CCTTGCAAGCTCTGCCTGTGGGGAATCCTGCTGAACCAAGCCTTATGATCGACGGAATTCTGTGGGAAGGTTT
CGGCGGAGATCCTTGCGATCCTTGCGCCACTTGGTGTGACGCTATCAGCATGCGTGTTGGTTACTACGGAGAC
TTTGTTTTCGACCGTGTTTTGAAAACTGATGTGAATAAAGAATTTCAGATGGGTGCCAAGCCTACAACTGATAC
AG--GCAATAGTGCA----
GCTCCATCCACTCTTACAGCAAGAGAGAATCCTGCTTACGGCCGACATATGCAGGATGCTGAGATGTTTACAA
ATGCCGCTTGCATGGCATTGAATATTTGGGATCGTTTTGATGTATTCTGTACATTAGGAGCCACCAGTGGATAT
CTTAAAGGAAACTCTGCTTCTTTCAATTTAGTTGGATTGTTTGGAGATAATGAAAATCAAAAAACGGTCAA---
AGCGGAGTCTGTACCAAATATGAGCTTTGATCAATCTGTTGTTGAGTTGTATACAGATACTACTTTTGCGTGGA
GCGTCGGCGCTCGCGCAGCTTTGTGGGAATGTGGATGTGCAACTTTAGGA

#CP_13
CCTTGCAAGCTCTGCCTGTGGGGAATCCTGCTGAACCAAGCCTTATGATCGACGGAATTCTGTGGGAAGGTTT
CGGCGGAGATCCTTGCGATCCTTGCACCACTTGGTGTGACGCTATCAGCATGCGTATGGGTTACTATGGTGACT
TTGTTTTCGACCGTGTTTTGAAAACAGATGTGAATAAAGAATTCCAAATGGGTGACAAGCCTACAAGTACTACA
G--GCAATGCTACA----
GCTCCAACCACTCTTACAGCAAGAGAGAATCCTGCTTACGGCCGACATATGCAGGATGCTGAGATGTTTACAA
ATGCCGCTTGCATGGCATTGAATATTTGGGATCGCTTTGATGTATTCTGTACACTAGGAGCCTCTAGCGGATAC
CTTAAAGGAAACTCTGCTTCTTTCAATTTAGTTGGATTGTTTGGAGATAATGAAAATCAAAGCACGGTCAA---
AACGAATTCTGTACCAAATATGAGCTTAGATCAATCTGTTGTTGAACTTTACACAGATACTGCCTTCTCTTGGAG
CGTGGGCGCTCGAGCAGCTTTGTGGGAGTGCGGATGTGCGACTTTAGGG

#CP_24
CCTTGCAAGCTCTGCCTGTGGGGAATCCTGCTGAACCAAGCCTTATGATCGACGGAATTCTGTGGGAAGGTTT
CGGCGGAGATCCTTGCGATCCTTGCACCACTTGGTGTGACGCTATCAGCATGCGTATGGGTTACTATGGTGACT
TTGTTTTCGACCGTGTTTTGAAAACAGATGTGAATAAAGAATTCCAAATGGGTGACAAGCCTACAAGTACTACA
G--GCAATGCTACA----
GCTCCAACCACTCTTACAGCAAGAGAGAATCCTGCTTACGGCCGACATATGCAGGATGCTGAGATGTTTACAA
ATGCCGCTTGCATGGCATTGAATATTTGGGATCGCTTTGATGTATTCTGTACACTAGGAGCCTCTAGCGGATAC
CTTAAAGGAAACTCTGCTTCTTTCAATTTAGTTGGATTGTTTGGAGATAATGAAAATCAAAGCACGGTCAA---
AACGAATTCTGTACCAAATATGAGCTTAGATCAATCTGTTGTTGAACTTTACACAGATACTGCCTTCTCTTGGAG
CGTGGGCGCTCGAGCAGCTTTGTGGGAGTGCGGATGTGCGACTTTAGGG

#CP_33
CCTTGCAAGCTCTGCCTGTGGGGAATCCTGCTGAACCAAGCCTTATGATCGACGGAATTCTGTGGGAAGGTTT
CGGCGGAGATCCTTGCGATCCTTGCACCACTTGGTGTGACGCTATCAGCATGCGTATGGGTTACTATGGTGACT
TTGTTTTCGACCGTGTTTTGAAAACAGATGTGAATAAAGAATTCCAAATGGGTGACAAGCCTACAAGTACTACA
G--GCAATGCTACA----
GCTCCAACCACTCTTACAGCAAGAGAGAATCCTGCTTACGGCCGACATATGCAGGATGCTGAGATGTTTACAA
ATGCCGCTTGCATGGCATTGAATATTTGGGATCGCTTTGATGTATTCTGTACACTAGGAGCCTCTAGCGGATAC
CTTAAAGGAAACTCTGCTTCTTTCAATTTAGTTGGATTGTTTGGAGATAATGAAAATCAAAGCACGGTCAA---
AACGAATTCTGTACCAAATATGAGCTTAGATCAATCTGTTGTTGAACTTTACACAGATACTGCCTTCTCTTGGAG
CGTGGGCGCTCGAGCAGCTTTGTGGGAGTGCGGATGTGCGACTTTAGGG

#CP_35
CCTTGCAAGCTCTGCCTGTGGGGAATCCTGCTGAACCAAGCCTTATGATCGACGGAATTCTGTGGGAAGGTTT
CGGCGGAGATCCTTGCGATCCTTGCACCACTTGGTGTGACGCTATCAGCATGCGTATGGGTTACTATGGTGACT
TTGTTTTCGACCGTGTTTTGAAAACAGATGTGAATAAAGAATTCCAAATGGGTGACAAGCCTACAAGTACTACA
G--GCAATGCTACA----
GCTCCAACCACTCTTACAGCAAGAGAGAATCCTGCTTACGGCCGACATATGCAGGATGCTGAGATGTTTACAA
ATGCCGCTTGCATGGCATTGAATATTTGGGATCGCTTTGATGTATTCTGTACACTAGGAGCCTCTAGCGGATAC
CTTAAAGGAAACTCTGCTTCTTTCAATTTAGTTGGATTGTTTGGAGATAATGAAAATCAAAGCACGGTCAA---
AACGAATTCTGTACCAAATATGAGCTTAGATCAATCTGTTGTTGAACTTTACACAGATACTGCCTTCTCTTGGAG
CGTGGGCGCTCGAGCAGCTTTGTGGGAGTGCGGATGTGCGACTTTAGGG

#CP_38_1
CCTTGCAAGCTCTGCCTGTGGGGAATCCTGCTGAACCAAGCCTTATGATCGACGGAATTCTGTGGGAAGGTTT
CGGCGGAGATCCTTGCGATCCTTGCACCACTTGGTGTGACGCTATCAGCATGCGTATGGGTTACTATGGTGACT
TTGTTTTCGACCGTGTTTTGAAAACAGATGTGAATAAAGAATTCCAAATGGGTGACAAGCCTACAAGTACTACA
G--GCAATGCTACA----
GCTCCAACCACTCTTACAGCAAGAGAGAATCCTGCTTACGGCCGACATATGCAGGATGCTGAGATGTTTACAA
ATGCCGCTTGCATGGCATTGAATATTTGGGATCGCTTTGATGTATTCTGTACACTAGGAGCCTCTAGCGGATAC
CTTAAAGGAAACTCTGCTTCTTTCAATTTAGTTGGATTGTTTGGAGATAATGAAAATCAAAGCACGGTCAA---
AACGAATTCTGTACCAAATATGAGCTTAGATCAATCTGTTGTTGAACTTTACACAGATACTGCCTTCTCTTGGAG
CGTGGGCGCTCGAGCAGCTTTGTGGGAGTGCGGATGTGCGACTTTAGGG

#CP_38_2
CCTTGCAAGCTCTGCCTGTGGGGAATCCTGCTGAACCAAGCCTTATGATCGACGGAATTCTGTGGGAAGGTTT
CGGCGGAGATCCTTGCGATCCTTGCACCACTTGGTGTGACGCTATCAGCATGCGTATGGGTTACTATGGTGACT
TTGTTTTCGACCGTGTTTTGAAAACAGATGTGAATAAAGAATTCCAAATGGGTGACAAGCCTACAAGTACTACA
G--GCAATGCTACA----
GCTCCAACCACTCTTACAGCAAGAGAGAATCCTGCTTACGGCCGACATATGCAGGATGCTGAGATGTTTACAA
ATGCCGCTTGCATGGCATTGAATATTTGGGATCGCTTTGATGTATTCTGTACACTAGGAGCCTCTAGCGGATAC
CTTAAAGGAAACTCTGCTTCTTTCAATTTAGTTGGATTGTTTGGAGATAATGAAAATCAAAGCACGGTCAA---
AACGAATTCTGTACCAAATATGAGCTTAGATCAATCTGTTGTTGAACTTTACACAGATACTGCCTTCTCTTGGAG
CGTGGGCGCTCGAGCAGCTTTGTGGGAGTGCGGATGTGCGACTTTAGGG

#CP_52
CCTTGCAAGCTCTGCCTGTGGGGAATCCTGCTGAACCAAGCCTTATGATCGACGGAATTCTGTGGGAAGGTTT
CGGCGGAGATCCTTGCGATCCTTGCACCACTTGGTGTGACGCTATCAGCATGCGTATGGGTTACTATGGTGACT
TTGTTTTCGACCGTGTTTTGAAAACAGATGTGAATAAAGAATTCCAAATGGGTGACAAGCCTACAAGTACTACA
G--GCAATGCTACA----
GCTCCAACCACTCTTACAGCAAGAGAGAATCCTGCTTACGGCCGACATATGCAGGATGCTGAGATGTTTACAA
ATGCCGCTTGCATGGCATTGAATATTTGGGATCGCTTTGATGTATTCTGTACACTAGGAGCCTCTAGCGGATAC
CTTAAAGGAAACTCTGCTTCTTTCAATTTAGTTGGATTGTTTGGAGATAATGAAAATCAAAGCACGGTCAA---
AACGAATTCTGTACCAAATATGAGCTTAGATCAATCTGTTGTTGAACTTTACACAGATACTGCCTTCTCTTGGAG
CGTGGGCGCTCGAGCAGCTTTGTGGGAGTGCGGATGTGCGACTTTAGGG

#CP_58_1
CCTTGCAAGCTCTGCCTGTGGGGAATCCTGCTGAACCAAGCCTTATGATCGACGGAATTCTGTGGGAAGGTTT
CGGCGGAGATCCTTGCGATCCTTGCACCACTTGGTGTGACGCTATCAGCATGCGTATGGGTTACTATGGTGACT
TTGTTTTCGACCGTGTTTTGAAAACAGATGTGAATAAAGAATTCCAAATGGGTGACAAGCCTACAAGTACTACA
G--GCAATGCTACA----
GCTCCAACCACTCTTACAGCAAGAGAGAATCCTGCTTACGGCCGACATATGCAGGATGCTGAGATGTTTACAA
ATGCCGCTTGCATGGCATTGAATATTTGGGATCGCTTTGATGTATTCTGTACACTAGGAGCCTCTAGCGGATAC
CTTAAAGGAAACTCTGCTTCTTTCAATTTAGTTGGATTGTTTGGAGATAATGAAAATCAAAGCACGGTCAA---
AACGAATTCTGTACCAAATATGAGCTTAGATCAATCTGTTGTTGAACTTTACACAGATACTGCCTTCTCTTGGAG
CGTGGGCGCTCGAGCAGCTTTGTGGGAGTGCGGATGTGCGACTTTAGGG

#CP_61
CCTTGCAAGCTCTGCCTGTGGGGAATCCTGCTGAACCAAGCCTTATGATCGACGGAATTCTGTGGGAAGGTTT
CGGCGGAGATCCTTGCGATCCTTGCACCACTTGGTGTGACGCTATCAGCATGCGTATGGGTTACTATGGTGACT
TTGTTTTCGACCGTGTTTTGAAAACAGATGTGAATAAAGAATTCCAAATGGGTGACAAGCCTACAAGTACTACA
G--GCAATGCTACA----
GCTCCAACCACTCTTACAGCAAGAGAGAATCCTGCTTACGGCCGACATATGCAGGATGCTGAGATGTTTACAA
ATGCCGCTTGCATGGCATTGAATATTTGGGATCGCTTTGATGTATTCTGTACACTAGGAGCCTCTAGCGGATAC
CTTAAAGGAAACTCTGCTTCTTTCAATTTAGTTGGATTGTTTGGAGATAATGAAAATCAAAGCACGGTCAA---
AACGAATTCTGTACCAAATATGAGCTTAGATCAATCTGTTGTTGAACTTTACACAGATACTGCCTTCTCTTGGAG
CGTGGGCGCTCGAGCAGCTTTGTGGGAGTGCGGATGTGCGACTTTAGGG

#CP_82
CCTTGCAAGCTCTGCCTGTGGGGAATCCTGCTGAACCAAGCCTTATGATCGACGGAATTCTGTGGGAAGGTTT
CGGCGGAGATCCTTGCGATCCTTGCACCACTTGGTGTGACGCTATCAGCATGCGTATGGGTTACTATGGTGACT
TTGTTTTCGACCGTGTTTTGAAAACAGATGTGAATAAAGAATTCCAAATGGGTGACAAGCCTACAAGTACTACA
G--GCAATGCTACA----
GCTCCAACCACTCTTACAGCAAGAGAGAATCCTGCTTACGGCCGACATATGCAGGATGCTGAGATGTTTACAA
ATGCCGCTTGCATGGCATTGAATATTTGGGATCGCTTTGATGTATTCTGTACACTAGGAGCCTCTAGCGGATAC
CTTAAAGGAAACTCTGCTTCTTTCAATTTAGTTGGATTGTTTGGAGATAATGAAAATCAAAGCACGGTCAA---
AACGAATTCTGTACCAAATATGAGCTTAGATCAATCTGTTGTTGAACTTTACACAGATACTGCCTTCTCTTGGAG
CGTGGGCGCTCGAGCAGCTTTGTGGGAGTGCGGATGTGCGACTTTAGGG

#CP_86
CCTTGCAAGCTCTGCCTGTGGGGAATCCTGCTGAACCAAGCCTTATGATCGACGGAATTCTGTGGGAAGGTTT
CGGCGGAGATCCTTGCGATCCTTGCACCACTTGGTGTGACGCTATCAGCATGCGTATGGGTTACTATGGTGACT
TTGTTTTCGACCGTGTTTTGAAAACAGATGTGAATAAAGAATTCCAAATGGGTGACAAGCCTACAAGTACTACA
G--GCAATGCTACA----
GCTCCAACCACTCTTACAGCAAGAGAGAATCCTGCTTACGGCCGACATATGCAGGATGCTGAGATGTTTACAA
ATGCCGCTTGCATGGCATTGAATATTTGGGATCGCTTTGATGTATTCTGTACACTAGGAGCCTCTAGCGGATAC
CTTAAAGGAAACTCTGCTTCTTTCAATTTAGTTGGATTGTTTGGAGATAATGAAAATCAAAGCACGGTCAA---
AACGAATTCTGTACCAAATATGAGCTTAGATCAATCTGTTGTTGAACTTTACACAGATACTGCCTTCTCTTGGAG
CGTGGGCGCTCGAGCAGCTTTGTGGGAGTGCGGATGTGCGACTTTAGGG

#CP_96
CCTTGCAAGCTCTGCCTGTGGGGAATCCTGCTGAACCAAGCCTTATGATCGACGGAATTCTGTGGGAAGGTTT
CGGCGGAGATCCTTGCGATCCTTGCACCACTTGGTGTGACGCTATCAGCATGCGTATGGGTTACTATGGTGACT
TTGTTTTCGACCGTGTTTTGAAAACAGATGTGAATAAAGAATTCCAAATGGGTGACAAGCCTACAAGTACTACA
G--GCAATGCTACA----
GCTCCAACCACTCTTACAGCAAGAGAGAATCCTGCTTACGGCCGACATATGCAGGATGCTGAGATGTTTACAA
ATGCCGCTTGCATGGCATTGAATATTTGGGATCGCTTTGATGTATTCTGTACACTAGGAGCCTCTAGCGGATAC
CTTAAAGGAAACTCTGCTTCTTTCAATTTAGTTGGATTGTTTGGAGATAATGAAAATCAAAGCACGGTCAA---
AACGAATTCTGTACCAAATATGAGCTTAGATCAATCTGTTGTTGAACTTTACACAGATACTGCCTTCTCTTGGAG
CGTGGGCGCTCGAGCAGCTTTGTGGGAGTGCGGATGTGCGACTTTAGGG

#CP_101
CCTTGCAAGCTCTGCCTGTGGGGAATCCTGCTGAACCAAGCCTTATGATCGACGGAATTCTGTGGGAAGGTTT
CGGCGGAGATCCTTGCGATCCTTGCACCACTTGGTGTGACGCTATCAGCATGCGTATGGGTTACTATGGTGACT
TTGTTTTCGACCGTGTTTTGAAAACAGATGTGAATAAAGAATTCCAAATGGGTGACAAGCCTACAAGTACTACA
G--GCAATGCTACA----
GCTCCAACCACTCTTACAGCAAGAGAGAATCCTGCTTACGGCCGACATATGCAGGATGCTGAGATGTTTACAA
ATGCCGCTTGCATGGCATTGAATATTTGGGATCGCTTTGATGTATTCTGTACACTAGGAGCCTCTAGCGGATAC
CTTAAAGGAAACTCTGCTTCTTTCAATTTAGTTGGATTGTTTGGAGATAATGAAAATCAAAGCACGGTCAA---
AACGAATTCTGTACCAAATATGAGCTTAGATCAATCTGTTGTTGAACTTTACACAGATACTGCCTTCTCTTGGAG
CGTGGGCGCTCGAGCAGCTTTGTGGGAGTGCGGATGTGCGACTTTAGGG

#CP_113
CCTTGCAAGCTCTGCCTGTGGGGAATCCTGCTGAACCAAGCCTTATGATCGACGGAATTCTGTGGGAAGGTTT
CGGCGGAGATCCTTGCGATCCTTGCACCACTTGGTGTGACGCTATCAGCATGCGTATGGGTTACTATGGTGACT
TTGTTTTCGACCGTGTTTTGAAAACAGATGTGAATAAAGAATTCCAAATGGGTGACAAGCCTACAAGTACTACA
G--GCAATGCTACA----
GCTCCAACCACTCTTACAGCAAGAGAGAATCCTGCTTACGGCCGACATATGCAGGATGCTGAGATGTTTACAA
ATGCCGCTTGCATGGCATTGAATATTTGGGATCGCTTTGATGTATTCTGTACACTAGGAGCCTCTAGCGGATAC
CTTAAAGGAAACTCTGCTTCTTTCAATTTAGTTGGATTGTTTGGAGATAATGAAAATCAAAGCACGGTCAA---
AACGAATTCTGTACCAAATATGAGCTTAGATCAATCTGTTGTTGAACTTTACACAGATACTGCCTTCTCTTGGAG
CGTGGGCGCTCGAGCAGCTTTGTGGGAGTGCGGATGTGCGACTTTAGGG

#CP_114
CCTTGCAAGCTCTGCCTGTGGGGAATCCTGCTGAACCAAGCCTTATGATCGACGGAATTCTGTGGGAAGGTTT
CGGCGGAGATCCTTGCGATCCTTGCACCACTTGGTGTGACGCTATCAGCATGCGTATGGGTTACTATGGTGACT
TTGTTTTCGACCGTGTTTTGAAAACAGATGTGAATAAAGAATTCCAAATGGGTGACAAGCCTACAAGTACTACA
G--GCAATGCTACA----
GCTCCAACCACTCTTACAGCAAGAGAGAATCCTGCTTACGGCCGACATATGCAGGATGCTGAGATGTTTACAA
ATGCCGCTTGCATGGCATTGAATATTTGGGATCGCTTTGATGTATTCTGTACACTAGGAGCCTCTAGCGGATAC
CTTAAAGGAAACTCTGCTTCTTTCAATTTAGTTGGATTGTTTGGAGATAATGAAAATCAAAGCACGGTCAA---
AACGAATTCTGTACCAAATATGAGCTTAGATCAATCTGTTGTTGAACTTTACACAGATACTGCCTTCTCTTGGAG
CGTGGGCGCTCGAGCAGCTTTGTGGGAGTGCGGATGTGCGACTTTAGGG

#CP_118
CCTTGCAAGCTCTGCCTGTGGGGAATCCTGCTGAACCAAGCCTTATGATCGACGGAATTCTGTGGGAAGGTTT
CGGCGGAGATCCTTGCGATCCTTGCACCACTTGGTGTGACGCTATCAGCATGCGTATGGGTTACTATGGTGACT
TTGTTTTCGACCGTGTTTTGAAAACAGATGTGAATAAAGAATTCCAAATGGGTGACAAGCCTACAAGTACTACA
G--GCAATGCTACA----
GCTCCAACCACTCTTACAGCAAGAGAGAATCCTGCTTACGGCCGACATATGCAGGATGCTGAGATGTTTACAA
ATGCCGCTTGCATGGCATTGAATATTTGGGATCGCTTTGATGTATTCTGTACACTAGGAGCCTCTAGCGGATAC
CTTAAAGGAAACTCTGCTTCTTTCAATTTAGTTGGATTGTTTGGAGATAATGAAAATCAAAGCACGGTCAA---
AACGAATTCTGTACCAAATATGAGCTTAGATCAATCTGTTGTTGAACTTTACACAGATACTGCCTTCTCTTGGAG
CGTGGGCGCTCGAGCAGCTTTGTGGGAGTGCGGATGTGCGACTTTAGGG

#CP_120_1
CCTTGCAAGCTCTGCCTGTGGGGAATCCTGCTGAACCAAGCCTTATGATCGACGGAATTCTGTGGGAAGGTTT
CGGCGGAGATCCTTGCGATCCTTGCACCACTTGGTGTGACGCTATCAGCATGCGTATGGGTTACTATGGTGACT
TTGTTTTCGACCGTGTTTTGAAAACAGATGTGAATAAAGAATTCCAAATGGGTGACAAGCCTACAAGTACTACA
G--GCAATGCTACA----
GCTCCAACCACTCTTACAGCAAGAGAGAATCCTGCTTACGGCCGACATATGCAGGATGCTGAGATGTTTACAA
ATGCCGCTTGCATGGCATTGAATATTTGGGATCGCTTTGATGTATTCTGTACACTAGGAGCCTCTAGCGGATAC
CTTAAAGGAAACTCTGCTTCTTTCAATTTAGTTGGATTGTTTGGAGATAATGAAAATCAAAGCACGGTCAA---
AACGAATTCTGTACCAAATATGAGCTTAGATCAATCTGTTGTTGAACTTTACACAGATACTGCCTTCTCTTGGAG
CGTGGGCGCTCGAGCAGCTTTGTGGGAGTGCGGATGTGCGACTTTAGGG

#CP_125
CCTTGCAAGCTCTGCCTGTGGGGAATCCTGCTGAACCAAGCCTTATGATCGACGGAATTCTGTGGGAAGGTTT
CGGCGGAGATCCTTGCGATCCTTGCACCACTTGGTGTGACGCTATCAGCATGCGTATGGGTTACTATGGTGACT
TTGTTTTCGACCGTGTTTTGAAAACAGATGTGAATAAAGAATTCCAAATGGGTGACAAGCCTACAAGTACTACA
G--GCAATGCTACA----
GCTCCAACCACTCTTACAGCAAGAGAGAATCCTGCTTACGGCCGACATATGCAGGATGCTGAGATGTTTACAA
ATGCCGCTTGCATGGCATTGAATATTTGGGATCGCTTTGATGTATTCTGTACACTAGGAGCCTCTAGCGGATAC
CTTAAAGGAAACTCTGCTTCTTTCAATTTAGTTGGATTGTTTGGAGATAATGAAAATCAAAGCACGGTCAA---
AACGAATTCTGTACCAAATATGAGCTTAGATCAATCTGTTGTTGAACTTTACACAGATACTGCCTTCTCTTGGAG
CGTGGGCGCTCGAGCAGCTTTGTGGGAGTGCGGATGTGCGACTTTAGGG

#CP_134
CCTTGCAAGCTCTGCCTGTGGGGAATCCTGCTGAACCAAGCCTTATGATCGACGGAATTCTGTGGGAAGGTTT
CGGCGGAGATCCTTGCGATCCTTGCACCACTTGGTGTGACGCTATCAGCATGCGTATGGGTTACTATGGTGACT
TTGTTTTCGACCGTGTTTTGAAAACAGATGTGAATAAAGAATTCCAAATGGGTGACAAGCCTACAAGTACTACA
G--GCAATGCTACA----
GCTCCAACCACTCTTACAGCAAGAGAGAATCCTGCTTACGGCCGACATATGCAGGATGCTGAGATGTTTACAA
ATGCCGCTTGCATGGCATTGAATATTTGGGATCGCTTTGATGTATTCTGTACACTAGGAGCCTCTAGCGGATAC
CTTAAAGGAAACTCTGCTTCTTTCAATTTAGTTGGATTGTTTGGAGATAATGAAAATCAAAGCACGGTCAA---
AACGAATTCTGTACCAAATATGAGCTTAGATCAATCTGTTGTTGAACTTTACACAGATACTGCCTTCTCTTGGAG
CGTGGGCGCTCGAGCAGCTTTGTGGGAGTGCGGATGTGCGACTTTAGGG

#CP_143
CCTTGCAAGCTCTGCCTGTGGGGAATCCTGCTGAACCAAGCCTTATGATCGACGGAATTCTGTGGGAAGGTTT
CGGCGGAGATCCTTGCGATCCTTGCACCACTTGGTGTGACGCTATCAGCATGCGTATGGGTTACTATGGTGACT
TTGTTTTCGACCGTGTTTTGAAAACAGATGTGAATAAAGAATTCCAAATGGGTGACAAGCCTACAAGTACTACA
G--GCAATGCTACA----
GCTCCAACCACTCTTACAGCAAGAGAGAATCCTGCTTACGGCCGACATATGCAGGATGCTGAGATGTTTACAA
ATGCCGCTTGCATGGCATTGAATATTTGGGATCGCTTTGATGTATTCTGTACACTAGGAGCCTCTAGCGGATAC
CTTAAAGGAAACTCTGCTTCTTTCAATTTAGTTGGATTGTTTGGAGATAATGAAAATCAAAGCACGGTCAA---
AACGAATTCTGTACCAAATATGAGCTTAGATCAATCTGTTGTTGAACTTTACACAGATACTGCCTTCTCTTGGAG
CGTGGGCGCTCGAGCAGCTTTGTGGGAGTGCGGATGTGCGACTTTAGGG

#CP_152
CCTTGCAAGCTCTGCCTGTGGGGAATCCTGCTGAACCAAGCCTTATGATCGACGGAATTCTGTGGGAAGGTTT
CGGCGGAGATCCTTGCGATCCTTGCACCACTTGGTGTGACGCTATCAGCATGCGTATGGGTTACTATGGTGACT
TTGTTTTCGACCGTGTTTTGAAAACAGATGTGAATAAAGAATTCCAAATGGGTGACAAGCCTACAAGTACTACA
G--GCAATGCTACA----
GCTCCAACCACTCTTACAGCAAGAGAGAATCCTGCTTACGGCCGACATATGCAGGATGCTGAGATGTTTACAA
ATGCCGCTTGCATGGCATTGAATATTTGGGATCGCTTTGATGTATTCTGTACACTAGGAGCCTCTAGCGGATAC
CTTAAAGGAAACTCTGCTTCTTTCAATTTAGTTGGATTGTTTGGAGATAATGAAAATCAAAGCACGGTCAA---
AACGAATTCTGTACCAAATATGAGCTTAGATCAATCTGTTGTTGAACTTTACACAGATACTGCCTTCTCTTGGAG
CGTGGGCGCTCGAGCAGCTTTGTGGGAGTGCGGATGTGCGACTTTAGGG

#CP_159
CCTTGCAAGCTCTGCCTGTGGGGAATCCTGCTGAACCAAGCCTTATGATCGACGGAATTCTGTGGGAAGGTTT
CGGCGGAGATCCTTGCGATCCTTGCACCACTTGGTGTGACGCTATCAGCATGCGTATGGGTTACTATGGTGACT
TTGTTTTCGACCGTGTTTTGAAAACAGATGTGAATAAAGAATTCCAAATGGGTGACAAGCCTACAAGTACTACA
G--GCAATGCTACA----
GCTCCAACCACTCTTACAGCAAGAGAGAATCCTGCTTACGGCCGACATATGCAGGATGCTGAGATGTTTACAA
ATGCCGCTTGCATGGCATTGAATATTTGGGATCGCTTTGATGTATTCTGTACACTAGGAGCCTCTAGCGGATAC
CTTAAAGGAAACTCTGCTTCTTTCAATTTAGTTGGATTGTTTGGAGATAATGAAAATCAAAGCACGGTCAA---
AACGAATTCTGTACCAAATATGAGCTTAGATCAATCTGTTGTTGAACTTTACACAGATACTGCCTTCTCTTGGAG
CGTGGGCGCTCGAGCAGCTTTGTGGGAGTGCGGATGTGCGACTTTAGGG

#CP_162
CCTTGCAAGCTCTGCCTGTGGGGAATCCTGCTGAACCAAGCCTTATGATCGACGGAATTCTGTGGGAAGGTTT
CGGCGGAGATCCTTGCGATCCTTGCACCACTTGGTGTGACGCTATCAGCATGCGTATGGGTTACTATGGTGACT
TTGTTTTCGACCGTGTTTTGAAAACAGATGTGAATAAAGAATTCCAAATGGGTGACAAGCCTACAAGTACTACA
G--GCAATGCTACA----
GCTCCAACCACTCTTACAGCAAGAGAGAATCCTGCTTACGGCCGACATATGCAGGATGCTGAGATGTTTACAA
ATGCCGCTTGCATGGCATTGAATATTTGGGATCGCTTTGATGTATTCTGTACACTAGGAGCCTCTAGCGGATAC
CTTAAAGGAAACTCTGCTTCTTTCAATTTAGTTGGATTGTTTGGAGATAATGAAAATCAAAGCACGGTCAA---
AACGAATTCTGTACCAAATATGAGCTTAGATCAATCTGTTGTTGAACTTTACACAGATACTGCCTTCTCTTGGAG
CGTGGGCGCTCGAGCAGCTTTGTGGGAGTGCGGATGTGCGACTTTAGGG

#CP_164
CCTTGCAAGCTCTGCCTGTGGGGAATCCTGCTGAACCAAGCCTTATGATCGACGGAATTCTGTGGGAAGGTTT
CGGCGGAGATCCTTGCGATCCTTGCACCACTTGGTGTGACGCTATCAGCATGCGTATGGGTTACTATGGTGACT
TTGTTTTCGACCGTGTTTTGAAAACAGATGTGAATAAAGAATTCCAAATGGGTGACAAGCCTACAAGTACTACA
G--GCAATGCTACA----
GCTCCAACCACTCTTACAGCAAGAGAGAATCCTGCTTACGGCCGACATATGCAGGATGCTGAGATGTTTACAA
ATGCCGCTTGCATGGCATTGAATATTTGGGATCGCTTTGATGTATTCTGTACACTAGGAGCCTCTAGCGGATAC
CTTAAAGGAAACTCTGCTTCTTTCAATTTAGTTGGATTGTTTGGAGATAATGAAAATCAAAGCACGGTCAA---
AACGAATTCTGTACCAAATATGAGCTTAGATCAATCTGTTGTTGAACTTTACACAGATACTGCCTTCTCTTGGAG
CGTGGGCGCTCGAGCAGCTTTGTGGGAGTGCGGATGTGCGACTTTAGGG

#CP_170
CCTTGCAAGCTCTGCCTGTGGGGAATCCTGCTGAACCAAGCCTTATGATCGACGGAATTCTGTGGGAAGGTTT
CGGCGGAGATCCTTGCGATCCTTGCACCACTTGGTGTGACGCTATCAGCATGCGTATGGGTTACTATGGTGACT
TTGTTTTCGACCGTGTTTTGAAAACAGATGTGAATAAAGAATTCCAAATGGGTGACAAGCCTACAAGTACTACA
G--GCAATGCTACA----
GCTCCAACCACTCTTACAGCAAGAGAGAATCCTGCTTACGGCCGACATATGCAGGATGCTGAGATGTTTACAA
ATGCCGCTTGCATGGCATTGAATATTTGGGATCGCTTTGATGTATTCTGTACACTAGGAGCCTCTAGCGGATAC
CTTAAAGGAAACTCTGCTTCTTTCAATTTAGTTGGATTGTTTGGAGATAATGAAAATCAAAGCACGGTCAA---
AACGAATTCTGTACCAAATATGAGCTTAGATCAATCTGTTGTTGAACTTTACACAGATACTGCCTTCTCTTGGAG
CGTGGGCGCTCGAGCAGCTTTGTGGGAGTGCGGATGTGCGACTTTAGGG

#CP_171
CCTTGCAAGCTCTGCCTGTGGGGAATCCTGCTGAACCAAGCCTTATGATCGACGGAATTCTGTGGGAAGGTTT
CGGCGGAGATCCTTGCGATCCTTGCACCACTTGGTGTGACGCTATCAGCATGCGTATGGGTTACTATGGTGACT
TTGTTTTCGACCGTGTTTTGAAAACAGATGTGAATAAAGAATTCCAAATGGGTGACAAGCCTACAAGTACTACA
G--GCAATGCTACA----
GCTCCAACCACTCTTACAGCAAGAGAGAATCCTGCTTACGGCCGACATATGCAGGATGCTGAGATGTTTACAA
ATGCCGCTTGCATGGCATTGAATATTTGGGATCGCTTTGATGTATTCTGTACACTAGGAGCCTCTAGCGGATAC
CTTAAAGGAAACTCTGCTTCTTTCAATTTAGTTGGATTGTTTGGAGATAATGAAAATCAAAGCACGGTCAA---
AACGAATTCTGTACCAAATATGAGCTTAGATCAATCTGTTGTTGAACTTTACACAGATACTGCCTTCTCTTGGAG
CGTGGGCGCTCGAGCAGCTTTGTGGGAGTGCGGATGTGCGACTTTAGGG

#CP_173
CCTTGCAAGCTCTGCCTGTGGGGAATCCTGCTGAACCAAGCCTTATGATCGACGGAATTCTGTGGGAAGGTTT
CGGCGGAGATCCTTGCGATCCTTGCACCACTTGGTGTGACGCTATCAGCATGCGTATGGGTTACTATGGTGACT
TTGTTTTCGACCGTGTTTTGAAAACAGATGTGAATAAAGAATTCCAAATGGGTGACAAGCCTACAAGTACTACA
G--GCAATGCTACA----
GCTCCAACCACTCTTACAGCAAGAGAGAATCCTGCTTACGGCCGACATATGCAGGATGCTGAGATGTTTACAA
ATGCCGCTTGCATGGCATTGAATATTTGGGATCGCTTTGATGTATTCTGTACACTAGGAGCCTCTAGCGGATAC
CTTAAAGGAAACTCTGCTTCTTTCAATTTAGTTGGATTGTTTGGAGATAATGAAAATCAAAGCACGGTCAA---
AACGAATTCTGTACCAAATATGAGCTTAGATCAATCTGTTGTTGAACTTTACACAGATACTGCCTTCTCTTGGAG
CGTGGGCGCTCGAGCAGCTTTGTGGGAGTGCGGATGTGCGACTTTAGGG

#CP_200
CCTTGCAAGCTCTGCCTGTGGGGAATCCTGCTGAACCAAGCCTTATGATCGACGGAATTCTGTGGGAAGGTTT
CGGCGGAGATCCTTGCGATCCTTGCACCACTTGGTGTGACGCTATCAGCATGCGTATGGGTTACTATGGTGACT
TTGTTTTCGACCGTGTTTTGAAAACAGATGTGAATAAAGAATTCCAAATGGGTGACAAGCCTACAAGTACTACA
G--GCAATGCTACA----
GCTCCAACCACTCTTACAGCAAGAGAGAATCCTGCTTACGGCCGACATATGCAGGATGCTGAGATGTTTACAA
ATGCCGCTTGCATGGCATTGAATATTTGGGATCGCTTTGATGTATTCTGTACACTAGGAGCCTCTAGCGGATAC
CTTAAAGGAAACTCTGCTTCTTTCAATTTAGTTGGATTGTTTGGAGATAATGAAAATCAAAGCACGGTCAA---
AACGAATTCTGTACCAAATATGAGCTTAGATCAATCTGTTGTTGAACTTTACACAGATACTGCCTTCTCTTGGAG
CGTGGGCGCTCGAGCAGCTTTGTGGGAGTGCGGATGTGCGACTTTAGGG

#CP_202
CCTTGCAAGCTCTGCCTGTGGGGAATCCTGCTGAACCAAGCCTTATGATCGACGGAATTCTGTGGGAAGGTTT
CGGCGGAGATCCTTGCGATCCTTGCACCACTTGGTGTGACGCTATCAGCATGCGTATGGGTTACTATGGTGACT
TTGTTTTCGACCGTGTTTTGAAAACAGATGTGAATAAAGAATTCCAAATGGGTGACAAGCCTACAAGTACTACA
G--GCAATGCTACA----
GCTCCAACCACTCTTACAGCAAGAGAGAATCCTGCTTACGGCCGACATATGCAGGATGCTGAGATGTTTACAA
ATGCCGCTTGCATGGCATTGAATATTTGGGATCGCTTTGATGTATTCTGTACACTAGGAGCCTCTAGCGGATAC
CTTAAAGGAAACTCTGCTTCTTTCAATTTAGTTGGATTGTTTGGAGATAATGAAAATCAAAGCACGGTCAA---
AACGAATTCTGTACCAAATATGAGCTTAGATCAATCTGTTGTTGAACTTTACACAGATACTGCCTTCTCTTGGAG
CGTGGGCGCTCGAGCAGCTTTGTGGGAGTGCGGATGTGCGACTTTAGGG

#CP_213_1
CCTTGCAAGCTCTGCCTGTGGGGAATCCTGCTGAACCAAGCCTTATGATCGACGGAATTCTGTGGGAAGGTTT
CGGCGGAGATCCTTGCGATCCTTGCACCACTTGGTGTGACGCTATCAGCATGCGTATGGGTTACTATGGTGACT
TTGTTTTCGACCGTGTTTTGAAAACAGATGTGAATAAAGAATTCCAAATGGGTGACAAGCCTACAAGTACTACA
G--GCAATGCTACA----
GCTCCAACCACTCTTACAGCAAGAGAGAATCCTGCTTACGGCCGACATATGCAGGATGCTGAGATGTTTACAA
ATGCCGCTTGCATGGCATTGAATATTTGGGATCGCTTTGATGTATTCTGTACACTAGGAGCCTCTAGCGGATAC
CTTAAAGGAAACTCTGCTTCTTTCAATTTAGTTGGATTGTTTGGAGATAATGAAAATCAAAGCACGGTCAA---
AACGAATTCTGTACCAAATATGAGCTTAGATCAATCTGTTGTTGAACTTTACACAGATACTGCCTTCTCTTGGAG
CGTGGGCGCTCGAGCAGCTTTGTGGGAGTGCGGATGTGCGACTTTAGGG

#CP_216
CCTTGCAAGCTCTGCCTGTGGGGAATCCTGCTGAACCAAGCCTTATGATCGACGGAATTCTGTGGGAAGGTTT
CGGCGGAGATCCTTGCGATCCTTGCACCACTTGGTGTGACGCTATCAGCATGCGTATGGGTTACTATGGTGACT
TTGTTTTCGACCGTGTTTTGAAAACAGATGTGAATAAAGAATTCCAAATGGGTGACAAGCCTACAAGTACTACA
G--GCAATGCTACA----
GCTCCAACCACTCTTACAGCAAGAGAGAATCCTGCTTACGGCCGACATATGCAGGATGCTGAGATGTTTACAA
ATGCCGCTTGCATGGCATTGAATATTTGGGATCGCTTTGATGTATTCTGTACACTAGGAGCCTCTAGCGGATAC
CTTAAAGGAAACTCTGCTTCTTTCAATTTAGTTGGATTGTTTGGAGATAATGAAAATCAAAGCACGGTCAA---
AACGAATTCTGTACCAAATATGAGCTTAGATCAATCTGTTGTTGAACTTTACACAGATACTGCCTTCTCTTGGAG
CGTGGGCGCTCGAGCAGCTTTGTGGGAGTGCGGATGTGCGACTTTAGGG

#CP_242
CCTTGCAAGCTCTGCCTGTGGGGAATCCTGCTGAACCAAGCCTTATGATCGACGGAATTCTGTGGGAAGGTTT
CGGCGGAGATCCTTGCGATCCTTGCACCACTTGGTGTGACGCTATCAGCATGCGTATGGGTTACTATGGTGACT
TTGTTTTCGACCGTGTTTTGAAAACAGATGTGAATAAAGAATTCCAAATGGGTGACAAGCCTACAAGTACTACA
G--GCAATGCTACA----
GCTCCAACCACTCTTACAGCAAGAGAGAATCCTGCTTACGGCCGACATATGCAGGATGCTGAGATGTTTACAA
ATGCCGCTTGCATGGCATTGAATATTTGGGATCGCTTTGATGTATTCTGTACACTAGGAGCCTCTAGCGGATAC
CTTAAAGGAAACTCTGCTTCTTTCAATTTAGTTGGATTGTTTGGAGATAATGAAAATCAAAGCACGGTCAA---
AACGAATTCTGTACCAAATATGAGCTTAGATCAATCTGTTGTTGAACTTTACACAGATACTGCCTTCTCTTGGAG
CGTGGGCGCTCGAGCAGCTTTGTGGGAGTGCGGATGTGCGACTTTAGGG

#CP_252
CCTTGCAAGCTCTGCCTGTGGGGAATCCTGCTGAACCAAGCCTTATGATCGACGGAATTCTGTGGGAAGGTTT
CGGCGGAGATCCTTGCGATCCTTGCACCACTTGGTGTGACGCTATCAGCATGCGTATGGGTTACTATGGTGACT
TTGTTTTCGACCGTGTTTTGAAAACAGATGTGAATAAAGAATTCCAAATGGGTGACAAGCCTACAAGTACTACA
G--GCAATGCTACA----
GCTCCAACCACTCTTACAGCAAGAGAGAATCCTGCTTACGGCCGACATATGCAGGATGCTGAGATGTTTACAA
ATGCCGCTTGCATGGCATTGAATATTTGGGATCGCTTTGATGTATTCTGTACACTAGGAGCCTCTAGCGGATAC
CTTAAAGGAAACTCTGCTTCTTTCAATTTAGTTGGATTGTTTGGAGATAATGAAAATCAAAGCACGGTCAA---
AACGAATTCTGTACCAAATATGAGCTTAGATCAATCTGTTGTTGAACTTTACACAGATACTGCCTTCTCTTGGAG
CGTGGGCGCTCGAGCAGCTTTGTGGGAGTGCGGATGTGCGACTTTAGGG

#CP_259
CCTTGCAAGCTCTGCCTGTGGGGAATCCTGCTGAACCAAGCCTTATGATCGACGGAATTCTGTGGGAAGGTTT
CGGCGGAGATCCTTGCGATCCTTGCACCACTTGGTGTGACGCTATCAGCATGCGTATGGGTTACTATGGTGACT
TTGTTTTCGACCGTGTTTTGAAAACAGATGTGAATAAAGAATTCCAAATGGGTGACAAGCCTACAAGTACTACA
G--GCAATGCTACA----
GCTCCAACCACTCTTACAGCAAGAGAGAATCCTGCTTACGGCCGACATATGCAGGATGCTGAGATGTTTACAA
ATGCCGCTTGCATGGCATTGAATATTTGGGATCGCTTTGATGTATTCTGTACACTAGGAGCCTCTAGCGGATAC
CTTAAAGGAAACTCTGCTTCTTTCAATTTAGTTGGATTGTTTGGAGATAATGAAAATCAAAGCACGGTCAA---
AACGAATTCTGTACCAAATATGAGCTTAGATCAATCTGTTGTTGAACTTTACACAGATACTGCCTTCTCTTGGAG
CGTGGGCGCTCGAGCAGCTTTGTGGGAGTGCGGATGTGCGACTTTAGGG

#CP_260_1
CCTTGCAAGCTCTGCCTGTGGGGAATCCTGCTGAACCAAGCCTTATGATCGACGGAATTCTGTGGGAAGGTTT
CGGCGGAGATCCTTGCGATCCTTGCACCACTTGGTGTGACGCTATCAGCATGCGTATGGGTTACTATGGTGACT
TTGTTTTCGACCGTGTTTTGAAAACAGATGTGAATAAAGAATTCCAAATGGGTGACAAGCCTACAAGTACTACA
G--GCAATGCTACA----
GCTCCAACCACTCTTACAGCAAGAGAGAATCCTGCTTACGGCCGACATATGCAGGATGCTGAGATGTTTACAA
ATGCCGCTTGCATGGCATTGAATATTTGGGATCGCTTTGATGTATTCTGTACACTAGGAGCCTCTAGCGGATAC
CTTAAAGGAAACTCTGCTTCTTTCAATTTAGTTGGATTGTTTGGAGATAATGAAAATCAAAGCACGGTCAA---
AACGAATTCTGTACCAAATATGAGCTTAGATCAATCTGTTGTTGAACTTTACACAGATACTGCCTTCTCTTGGAG
CGTGGGCGCTCGAGCAGCTTTGTGGGAGTGCGGATGTGCGACTTTAGGG

#CP_260_2
CCTTGCAAGCTCTGCCTGTGGGGAATCCTGCTGAACCAAGCCTTATGATCGACGGAATTCTGTGGGAAGGTTT
CGGCGGAGATCCTTGCGATCCTTGCACCACTTGGTGTGACGCTATCAGCATGCGTATGGGTTACTATGGTGACT
TTGTTTTCGACCGTGTTTTGAAAACAGATGTGAATAAAGAATTCCAAATGGGTGACAAGCCTACAAGTACTACA
G--GCAATGCTACA----
GCTCCAACCACTCTTACAGCAAGAGAGAATCCTGCTTACGGCCGACATATGCAGGATGCTGAGATGTTTACAA
ATGCCGCTTGCATGGCATTGAATATTTGGGATCGCTTTGATGTATTCTGTACACTAGGAGCCTCTAGCGGATAC
CTTAAAGGAAACTCTGCTTCTTTCAATTTAGTTGGATTGTTTGGAGATAATGAAAATCAAAGCACGGTCAA---
AACGAATTCTGTACCAAATATGAGCTTAGATCAATCTGTTGTTGAACTTTACACAGATACTGCCTTCTCTTGGAG
CGTGGGCGCTCGAGCAGCTTTGTGGGAGTGCGGATGTGCGACTTTAGGG

#CP_267
CCTTGCAAGCTCTGCCTGTGGGGAATCCTGCTGAACCAAGCCTTATGATCGACGGAATTCTGTGGGAAGGTTT
CGGCGGAGATCCTTGCGATCCTTGCACCACTTGGTGTGACGCTATCAGCATGCGTATGGGTTACTATGGTGACT
TTGTTTTCGACCGTGTTTTGAAAACAGATGTGAATAAAGAATTCCAAATGGGTGACAAGCCTACAAGTACTACA
G--GCAATGCTACA----
GCTCCAACCACTCTTACAGCAAGAGAGAATCCTGCTTACGGCCGACATATGCAGGATGCTGAGATGTTTACAA
ATGCCGCTTGCATGGCATTGAATATTTGGGATCGCTTTGATGTATTCTGTACACTAGGAGCCTCTAGCGGATAC
CTTAAAGGAAACTCTGCTTCTTTCAATTTAGTTGGATTGTTTGGAGATAATGAAAATCAAAGCACGGTCAA---
AACGAATTCTGTACCAAATATGAGCTTAGATCAATCTGTTGTTGAACTTTACACAGATACTGCCTTCTCTTGGAG
CGTGGGCGCTCGAGCAGCTTTGTGGGAGTGCGGATGTGCGACTTTAGGG

#CP_269
CCTTGCAAGCTCTGCCTGTGGGGAATCCTGCTGAACCAAGCCTTATGATCGACGGAATTCTGTGGGAAGGTTT
CGGCGGAGATCCTTGCGATCCTTGCACCACTTGGTGTGACGCTATCAGCATGCGTATGGGTTACTATGGTGACT
TTGTTTTCGACCGTGTTTTGAAAACAGATGTGAATAAAGAATTCCAAATGGGTGACAAGCCTACAAGTACTACA
G--GCAATGCTACA----
GCTCCAACCACTCTTACAGCAAGAGAGAATCCTGCTTACGGCCGACATATGCAGGATGCTGAGATGTTTACAA
ATGCCGCTTGCATGGCATTGAATATTTGGGATCGCTTTGATGTATTCTGTACACTAGGAGCCTCTAGCGGATAC
CTTAAAGGAAACTCTGCTTCTTTCAATTTAGTTGGATTGTTTGGAGATAATGAAAATCAAAGCACGGTCAA---
AACGAATTCTGTACCAAATATGAGCTTAGATCAATCTGTTGTTGAACTTTACACAGATACTGCCTTCTCTTGGAG
CGTGGGCGCTCGAGCAGCTTTGTGGGAGTGCGGATGTGCGACTTTAGGG

#CP_275
CCTTGCAAGCTCTGCCTGTGGGGAATCCTGCTGAACCAAGCCTTATGATCGACGGAATTCTGTGGGAAGGTTT
CGGCGGAGATCCTTGCGATCCTTGCACCACTTGGTGTGACGCTATCAGCATGCGTATGGGTTACTATGGTGACT
TTGTTTTCGACCGTGTTTTGAAAACAGATGTGAATAAAGAATTCCAAATGGGTGACAAGCCTACAAGTACTACA
G--GCAATGCTACA----
GCTCCAACCACTCTTACAGCAAGAGAGAATCCTGCTTACGGCCGACATATGCAGGATGCTGAGATGTTTACAA
ATGCCGCTTGCATGGCATTGAATATTTGGGATCGCTTTGATGTATTCTGTACACTAGGAGCCTCTAGCGGATAC
CTTAAAGGAAACTCTGCTTCTTTCAATTTAGTTGGATTGTTTGGAGATAATGAAAATCAAAGCACGGTCAA---
AACGAATTCTGTACCAAATATGAGCTTAGATCAATCTGTTGTTGAACTTTACACAGATACTGCCTTCTCTTGGAG
CGTGGGCGCTCGAGCAGCTTTGTGGGAGTGCGGATGTGCGACTTTAGGG

#CP_301
CCTTGCAAGCTCTGCCTGTGGGGAATCCTGCTGAACCAAGCCTTATGATCGACGGAATTCTGTGGGAAGGTTT
CGGCGGAGATCCTTGCGATCCTTGCACCACTTGGTGTGACGCTATCAGCATGCGTATGGGTTACTATGGTGACT
TTGTTTTCGACCGTGTTTTGAAAACAGATGTGAATAAAGAATTCCAAATGGGTGACAAGCCTACAAGTACTACA
G--GCAATGCTACA----
GCTCCAACCACTCTTACAGCAAGAGAGAATCCTGCTTACGGCCGACATATGCAGGATGCTGAGATGTTTACAA
ATGCCGCTTGCATGGCATTGAATATTTGGGATCGCTTTGATGTATTCTGTACACTAGGAGCCTCTAGCGGATAC
CTTAAAGGAAACTCTGCTTCTTTCAATTTAGTTGGATTGTTTGGAGATAATGAAAATCAAAGCACGGTCAA---
AACGAATTCTGTACCAAATATGAGCTTAGATCAATCTGTTGTTGAACTTTACACAGATACTGCCTTCTCTTGGAG
CGTGGGCGCTCGAGCAGCTTTGTGGGAGTGCGGATGTGCGACTTTAGGG

#CP_310
CCTTGCAAGCTCTGCCTGTGGGGAATCCTGCTGAACCAAGCCTTATGATCGACGGAATTCTGTGGGAAGGTTT
CGGCGGAGATCCTTGCGATCCTTGCACCACTTGGTGTGACGCTATCAGCATGCGTATGGGTTACTATGGTGACT
TTGTTTTCGACCGTGTTTTGAAAACAGATGTGAATAAAGAATTCCAAATGGGTGACAAGCCTACAAGTACTACA
G--GCAATGCTACA----
GCTCCAACCACTCTTACAGCAAGAGAGAATCCTGCTTACGGCCGACATATGCAGGATGCTGAGATGTTTACAA
ATGCCGCTTGCATGGCATTGAATATTTGGGATCGCTTTGATGTATTCTGTACACTAGGAGCCTCTAGCGGATAC
CTTAAAGGAAACTCTGCTTCTTTCAATTTAGTTGGATTGTTTGGAGATAATGAAAATCAAAGCACGGTCAA---
AACGAATTCTGTACCAAATATGAGCTTAGATCAATCTGTTGTTGAACTTTACACAGATACTGCCTTCTCTTGGAG
CGTGGGCGCTCGAGCAGCTTTGTGGGAGTGCGGATGTGCGACTTTAGGG

#CP_311
CCTTGCAAGCTCTGCCTGTGGGGAATCCTGCTGAACCAAGCCTTATGATCGACGGAATTCTGTGGGAAGGTTT
CGGCGGAGATCCTTGCGATCCTTGCACCACTTGGTGTGACGCTATCAGCATGCGTATGGGTTACTATGGTGACT
TTGTTTTCGACCGTGTTTTGAAAACAGATGTGAATAAAGAATTCCAAATGGGTGACAAGCCTACAAGTACTACA
G--GCAATGCTACA----
GCTCCAACCACTCTTACAGCAAGAGAGAATCCTGCTTACGGCCGACATATGCAGGATGCTGAGATGTTTACAA
ATGCCGCTTGCATGGCATTGAATATTTGGGATCGCTTTGATGTATTCTGTACACTAGGAGCCTCTAGCGGATAC
CTTAAAGGAAACTCTGCTTCTTTCAATTTAGTTGGATTGTTTGGAGATAATGAAAATCAAAGCACGGTCAA---
AACGAATTCTGTACCAAATATGAGCTTAGATCAATCTGTTGTTGAACTTTACACAGATACTGCCTTCTCTTGGAG
CGTGGGCGCTCGAGCAGCTTTGTGGGAGTGCGGATGTGCGACTTTAGGG

#CP_314
CCTTGCAAGCTCTGCCTGTGGGGAATCCTGCTGAACCAAGCCTTATGATCGACGGAATTCTGTGGGAAGGTTT
CGGCGGAGATCCTTGCGATCCTTGCACCACTTGGTGTGACGCTATCAGCATGCGTATGGGTTACTATGGTGACT
TTGTTTTCGACCGTGTTTTGAAAACAGATGTGAATAAAGAATTCCAAATGGGTGACAAGCCTACAAGTACTACA
G--GCAATGCTACA----
GCTCCAACCACTCTTACAGCAAGAGAGAATCCTGCTTACGGCCGACATATGCAGGATGCTGAGATGTTTACAA
ATGCCGCTTGCATGGCATTGAATATTTGGGATCGCTTTGATGTATTCTGTACACTAGGAGCCTCTAGCGGATAC
CTTAAAGGAAACTCTGCTTCTTTCAATTTAGTTGGATTGTTTGGAGATAATGAAAATCAAAGCACGGTCAA---
AACGAATTCTGTACCAAATATGAGCTTAGATCAATCTGTTGTTGAACTTTACACAGATACTGCCTTCTCTTGGAG
CGTGGGCGCTCGAGCAGCTTTGTGGGAGTGCGGATGTGCGACTTTAGGG

#CP_317

CCTTGCAAGCTCTGCCTGTGGGGAATCCTGCTGAACCAAGCCTTATGATCGACGGAATTCTGTGGGAAGGTTT
CGGCGGAGATCCTTGCGATCCTTGCACCACTTGGTGTGACGCTATCAGCATGCGTATGGGTTACTATGGTGACT
TTGTTTTCGACCGTGTTTTGAAAACAGATGTGAATAAAGAATTCCAAATGGGTGACAAGCCTACAAGTACTACA
G--GCAATGCTACA----
GCTCCAACCACTCTTACAGCAAGAGAGAATCCTGCTTACGGCCGACATATGCAGGATGCTGAGATGTTTACAA
ATGCCGCTTGCATGGCATTGAATATTTGGGATCGCTTTGATGTATTCTGTACACTAGGAGCCTCTAGCGGATAC
CTTAAAGGAAACTCTGCTTCTTTCAATTTAGTTGGATTGTTTGGAGATAATGAAAATCAAAGCACGGTCAA---
AACGAATTCTGTACCAAATATGAGCTTAGATCAATCTGTTGTTGAACTTTACACAGATACTGCCTTCTCTTGGAG
CGTGGGCGCTCGAGCAGCTTTGTGGGAGTGCGGATGTGCGACTTTAGGG

#CP_342

CCTTGCAAGCTCTGCCTGTGGGGAATCCTGCTGAACCAAGCCTTATGATCGACGGAATTCTGTGGGAAGGTTT
CGGCGGAGATCCTTGCGATCCTTGCACCACTTGGTGTGACGCTATCAGCATGCGTATGGGTTACTATGGTGACT
TTGTTTTCGACCGTGTTTTGAAAACAGATGTGAATAAAGAATTCCAAATGGGTGACAAGCCTACAAGTACTACA
G--GCAATGCTACA----
GCTCCAACCACTCTTACAGCAAGAGAGAATCCTGCTTACGGCCGACATATGCAGGATGCTGAGATGTTTACAA
ATGCCGCTTGCATGGCATTGAATATTTGGGATCGCTTTGATGTATTCTGTACACTAGGAGCCTCTAGCGGATAC
CTTAAAGGAAACTCTGCTTCTTTCAATTTAGTTGGATTGTTTGGAGATAATGAAAATCAAAGCACGGTCAA---
AACGAATTCTGTACCAAATATGAGCTTAGATCAATCTGTTGTTGAACTTTACACAGATACTGCCTTCTCTTGGAG
CGTGGGCGCTCGAGCAGCTTTGTGGGAGTGCGGATGTGCGACTTTAGGG

#CP_344

CCTTGCAAGCTCTGCCTGTGGGGAATCCTGCTGAACCAAGCCTTATGATCGACGGAATTCTGTGGGAAGGTTT
CGGCGGAGATCCTTGCGATCCTTGCACCACTTGGTGTGACGCTATCAGCATGCGTATGGGTTACTATGGTGACT
TTGTTTTCGACCGTGTTTTGAAAACAGATGTGAATAAAGAATTCCAAATGGGTGACAAGCCTACAAGTACTACA
G--GCAATGCTACA----
GCTCCAACCACTCTTACAGCAAGAGAGAATCCTGCTTACGGCCGACATATGCAGGATGCTGAGATGTTTACAA
ATGCCGCTTGCATGGCATTGAATATTTGGGATCGCTTTGATGTATTCTGTACACTAGGAGCCTCTAGCGGATAC
CTTAAAGGAAACTCTGCTTCTTTCAATTTAGTTGGATTGTTTGGAGATAATGAAAATCAAAGCACGGTCAA---
AACGAATTCTGTACCAAATATGAGCTTAGATCAATCTGTTGTTGAACTTTACACAGATACTGCCTTCTCTTGGAG
CGTGGGCGCTCGAGCAGCTTTGTGGGAGTGCGGATGTGCGACTTTAGGG

#CP_348

CCTTGCAAGCTCTGCCTGTGGGGAATCCTGCTGAACCAAGCCTTATGATCGACGGAATTCTGTGGGAAGGTTT
CGGCGGAGATCCTTGCGATCCTTGCACCACTTGGTGTGACGCTATCAGCATGCGTATGGGTTACTATGGTGACT
TTGTTTTCGACCGTGTTTTGAAAACAGATGTGAATAAAGAATTCCAAATGGGTGACAAGCCTACAAGTACTACA
G--GCAATGCTACA----
GCTCCAACCACTCTTACAGCAAGAGAGAATCCTGCTTACGGCCGACATATGCAGGATGCTGAGATGTTTACAA
ATGCCGCTTGCATGGCATTGAATATTTGGGATCGCTTTGATGTATTCTGTACACTAGGAGCCTCTAGCGGATAC
CTTAAAGGAAACTCTGCTTCTTTCAATTTAGTTGGATTGTTTGGAGATAATGAAAATCAAAGCACGGTCAA---
AACGAATTCTGTACCAAATATGAGCTTAGATCAATCTGTTGTTGAACTTTACACAGATACTGCCTTCTCTTGGAG
CGTGGGCGCTCGAGCAGCTTTGTGGGAGTGCGGATGTGCGACTTTAGGG

#CP_351
CCTTGCAAGCTCTGCCTGTGGGGAATCCTGCTGAACCAAGCCTTATGATCGACGGAATTCTGTGGGAAGGTTT
CGGCGGAGATCCTTGCGATCCTTGCACCACTTGGTGTGACGCTATCAGCATGCGTATGGGTTACTATGGTGACT
TTGTTTTCGACCGTGTTTTGAAAACAGATGTGAATAAAGAATTCCAAATGGGTGACAAGCCTACAAGTACTACA
G--GCAATGCTACA----
GCTCCAACCACTCTTACAGCAAGAGAGAATCCTGCTTACGGCCGACATATGCAGGATGCTGAGATGTTTACAA
ATGCCGCTTGCATGGCATTGAATATTTGGGATCGCTTTGATGTATTCTGTACACTAGGAGCCTCTAGCGGATAC
CTTAAAGGAAACTCTGCTTCTTTCAATTTAGTTGGATTGTTTGGAGATAATGAAAATCAAAGCACGGTCAA---
AACGAATTCTGTACCAAATATGAGCTTAGATCAATCTGTTGTTGAACTTTACACAGATACTGCCTTCTCTTGGAG
CGTGGGCGCTCGAGCAGCTTTGTGGGAGTGCGGATGTGCGACTTTAGGG

#CP_353
CCTTGCAAGCTCTGCCTGTGGGGAATCCTGCTGAACCAAGCCTTATGATCGACGGAATTCTGTGGGAAGGTTT
CGGCGGAGATCCTTGCGATCCTTGCACCACTTGGTGTGACGCTATCAGCATGCGTATGGGTTACTATGGTGACT
TTGTTTTCGACCGTGTTTTGAAAACAGATGTGAATAAAGAATTCCAAATGGGTGACAAGCCTACAAGTACTACA
G--GCAATGCTACA----
GCTCCAACCACTCTTACAGCAAGAGAGAATCCTGCTTACGGCCGACATATGCAGGATGCTGAGATGTTTACAA
ATGCCGCTTGCATGGCATTGAATATTTGGGATCGCTTTGATGTATTCTGTACACTAGGAGCCTCTAGCGGATAC
CTTAAAGGAAACTCTGCTTCTTTCAATTTAGTTGGATTGTTTGGAGATAATGAAAATCAAAGCACGGTCAA---
AACGAATTCTGTACCAAATATGAGCTTAGATCAATCTGTTGTTGAACTTTACACAGATACTGCCTTCTCTTGGAG
CGTGGGCGCTCGAGCAGCTTTGTGGGAGTGCGGATGTGCGACTTTAGGG

#CP_371
CCTTGCAAGCTCTGCCTGTGGGGAATCCTGCTGAACCAAGCCTTATGATCGACGGAATTCTGTGGGAAGGTTT
CGGCGGAGATCCTTGCGATCCTTGCACCACTTGGTGTGACGCTATCAGCATGCGTATGGGTTACTATGGTGACT
TTGTTTTCGACCGTGTTTTGAAAACAGATGTGAATAAAGAATTCCAAATGGGTGACAAGCCTACAAGTACTACA
G--GCAATGCTACA----
GCTCCAACCACTCTTACAGCAAGAGAGAATCCTGCTTACGGCCGACATATGCAGGATGCTGAGATGTTTACAA
ATGCCGCTTGCATGGCATTGAATATTTGGGATCGCTTTGATGTATTCTGTACACTAGGAGCCTCTAGCGGATAC
CTTAAAGGAAACTCTGCTTCTTTCAATTTAGTTGGATTGTTTGGAGATAATGAAAATCAAAGCACGGTCAA---
AACGAATTCTGTACCAAATATGAGCTTAGATCAATCTGTTGTTGAACTTTACACAGATACTGCCTTCTCTTGGAG
CGTGGGCGCTCGAGCAGCTTTGTGGGAGTGCGGATGTGCGACTTTAGGG

#CP_376
CCTTGCAAGCTCTGCCTGTGGGGAATCCTGCTGAACCAAGCCTTATGATCGACGGAATTCTGTGGGAAGGTTT
CGGCGGAGATCCTTGCGATCCTTGCACCACTTGGTGTGACGCTATCAGCATGCGTATGGGTTACTATGGTGACT
TTGTTTTCGACCGTGTTTTGAAAACAGATGTGAATAAAGAATTCCAAATGGGTGACAAGCCTACAAGTACTACA
G--GCAATGCTACA----
GCTCCAACCACTCTTACAGCAAGAGAGAATCCTGCTTACGGCCGACATATGCAGGATGCTGAGATGTTTACAA
ATGCCGCTTGCATGGCATTGAATATTTGGGATCGCTTTGATGTATTCTGTACACTAGGAGCCTCTAGCGGATAC
CTTAAAGGAAACTCTGCTTCTTTCAATTTAGTTGGATTGTTTGGAGATAATGAAAATCAAAGCACGGTCAA---
AACGAATTCTGTACCAAATATGAGCTTAGATCAATCTGTTGTTGAACTTTACACAGATACTGCCTTCTCTTGGAG
CGTGGGCGCTCGAGCAGCTTTGTGGGAGTGCGGATGTGCGACTTTAGGG

#CP_381
CCTTGCAAGCTCTGCCTGTGGGGAATCCTGCTGAACCAAGCCTTATGATCGACGGAATTCTGTGGGAAGGTTT
CGGCGGAGATCCTTGCGATCCTTGCACCACTTGGTGTGACGCTATCAGCATGCGTATGGGTTACTATGGTGACT
TTGTTTTCGACCGTGTTTTGAAAACAGATGTGAATAAAGAATTCCAAATGGGTGACAAGCCTACAAGTACTACA
G--GCAATGCTACA----
GCTCCAACCACTCTTACAGCAAGAGAGAATCCTGCTTACGGCCGACATATGCAGGATGCTGAGATGTTTACAA
ATGCCGCTTGCATGGCATTGAATATTTGGGATCGCTTTGATGTATTCTGTACACTAGGAGCCTCTAGCGGATAC
CTTAAAGGAAACTCTGCTTCTTTCAATTTAGTTGGATTGTTTGGAGATAATGAAAATCAAAGCACGGTCAA---
AACGAATTCTGTACCAAATATGAGCTTAGATCAATCTGTTGTTGAACTTTACACAGATACTGCCTTCTCTTGGAG
CGTGGGCGCTCGAGCAGCTTTGTGGGAGTGCGGATGTGCGACTTTAGGG

#CP_382
CCTTGCAAGCTCTGCCTGTGGGGAATCCTGCTGAACCAAGCCTTATGATCGACGGAATTCTGTGGGAAGGTTT
CGGCGGAGATCCTTGCGATCCTTGCACCACTTGGTGTGACGCTATCAGCATGCGTATGGGTTACTATGGTGACT
TTGTTTTCGACCGTGTTTTGAAAACAGATGTGAATAAAGAATTCCAAATGGGTGACAAGCCTACAAGTACTACA
G--GCAATGCTACA----
GCTCCAACCACTCTTACAGCAAGAGAGAATCCTGCTTACGGCCGACATATGCAGGATGCTGAGATGTTTACAA
ATGCCGCTTGCATGGCATTGAATATTTGGGATCGCTTTGATGTATTCTGTACACTAGGAGCCTCTAGCGGATAC
CTTAAAGGAAACTCTGCTTCTTTCAATTTAGTTGGATTGTTTGGAGATAATGAAAATCAAAGCACGGTCAA---
AACGAATTCTGTACCAAATATGAGCTTAGATCAATCTGTTGTTGAACTTTACACAGATACTGCCTTCTCTTGGAG
CGTGGGCGCTCGAGCAGCTTTGTGGGAGTGCGGATGTGCGACTTTAGGG

#CP_400
CCTTGCAAGCTCTGCCTGTGGGGAATCCTGCTGAACCAAGCCTTATGATCGACGGAATTCTGTGGGAAGGTTT
CGGCGGAGATCCTTGCGATCCTTGCACCACTTGGTGTGACGCTATCAGCATGCGTATGGGTTACTATGGTGACT
TTGTTTTCGACCGTGTTTTGAAAACAGATGTGAATAAAGAATTCCAAATGGGTGACAAGCCTACAAGTACTACA
G--GCAATGCTACA----
GCTCCAACCACTCTTACAGCAAGAGAGAATCCTGCTTACGGCCGACATATGCAGGATGCTGAGATGTTTACAA
ATGCCGCTTGCATGGCATTGAATATTTGGGATCGCTTTGATGTATTCTGTACACTAGGAGCCTCTAGCGGATAC
CTTAAAGGAAACTCTGCTTCTTTCAATTTAGTTGGATTGTTTGGAGATAATGAAAATCAAAGCACGGTCAA---
AACGAATTCTGTACCAAATATGAGCTTAGATCAATCTGTTGTTGAACTTTACACAGATACTGCCTTCTCTTGGAG
CGTGGGCGCTCGAGCAGCTTTGTGGGAGTGCGGATGTGCGACTTTAGGG

#CP_421
CCTTGCAAGCTCTGCCTGTGGGGAATCCTGCTGAACCAAGCCTTATGATCGACGGAATTCTGTGGGAAGGTTT
CGGCGGAGATCCTTGCGATCCTTGCACCACTTGGTGTGACGCTATCAGCATGCGTATGGGTTACTATGGTGACT
TTGTTTTCGACCGTGTTTTGAAAACAGATGTGAATAAAGAATTCCAAATGGGTGACAAGCCTACAAGTACTACA
G--GCAATGCTACA----
GCTCCAACCACTCTTACAGCAAGAGAGAATCCTGCTTACGGCCGACATATGCAGGATGCTGAGATGTTTACAA
ATGCCGCTTGCATGGCATTGAATATTTGGGATCGCTTTGATGTATTCTGTACACTAGGAGCCTCTAGCGGATAC
CTTAAAGGAAACTCTGCTTCTTTCAATTTAGTTGGATTGTTTGGAGATAATGAAAATCAAAGCACGGTCAA---
AACGAATTCTGTACCAAATATGAGCTTAGATCAATCTGTTGTTGAACTTTACACAGATACTGCCTTCTCTTGGAG
CGTGGGCGCTCGAGCAGCTTTGTGGGAGTGCGGATGTGCGACTTTAGGG

#CP_423
CCTTGCAAGCTCTGCCTGTGGGGAATCCTGCTGAACCAAGCCTTATGATCGACGGAATTCTGTGGGAAGGTTT
CGGCGGAGATCCTTGCGATCCTTGCACCACTTGGTGTGACGCTATCAGCATGCGTATGGGTTACTATGGTGACT
TTGTTTTCGACCGTGTTTTGAAAACAGATGTGAATAAAGAATTCCAAATGGGTGACAAGCCTACAAGTACTACA
G--GCAATGCTACA----
GCTCCAACCACTCTTACAGCAAGAGAGAATCCTGCTTACGGCCGACATATGCAGGATGCTGAGATGTTTACAA
ATGCCGCTTGCATGGCATTGAATATTTGGGATCGCTTTGATGTATTCTGTACACTAGGAGCCTCTAGCGGATAC
CTTAAAGGAAACTCTGCTTCTTTCAATTTAGTTGGATTGTTTGGAGATAATGAAAATCAAAGCACGGTCAA---
AACGAATTCTGTACCAAATATGAGCTTAGATCAATCTGTTGTTGAACTTTACACAGATACTGCCTTCTCTTGGAG
CGTGGGCGCTCGAGCAGCTTTGTGGGAGTGCGGATGTGCGACTTTAGGG

#FSW_1
CCTTGCAAGCTCTGCCTGTGGGGAATCCTGCTGAACCAAGCCTTATGATCGACGGAATTCTGTGGGAAGGTTT
CGGCGGAGATCCTTGCGATCCTTGCACCACTTGGTGTGACGCTATCAGCATGCGTATGGGTTACTATGGTGACT
TTGTTTTCGACCGTGTTTTGAAAACAGATGTGAATAAAGAATTCCAAATGGGTGACAAGCCTACAAGTACTACA
G--GCAATGCTACA----
GCTCCAACCACTCTTACAGCAAGAGAGAATCCTGCTTACGGCCGACATATGCAGGATGCTGAGATGTTTACAA
ATGCCGCTTGCATGGCATTGAATATTTGGGATCGCTTTGATGTATTCTGTACACTAGGAGCCTCTAGCGGATAC
CTTAAAGGAAACTCTGCTTCTTTCAATTTAGTTGGATTGTTTGGAGATAATGAAAATCAAAGCACGGTCAA---
AACGAATTCTGTACCAAATATGAGCTTAGATCAATCTGTTGTTGAACTTTACACAGATACTGCCTTCTCTTGGAG
CGTGGGCGCTCGAGCAGCTTTGTGGGAGTGCGGATGTGCGACTTTAGGG

#FSW_5
CCTTGCAAGCTCTGCCTGTGGGGAATCCTGCTGAACCAAGCCTTATGATCGACGGAATTCTGTGGGAAGGTTT
CGGCGGAGATCCTTGCGATCCTTGCACCACTTGGTGTGACGCTATCAGCATGCGTATGGGTTACTATGGTGACT
TTGTTTTCGACCGTGTTTTGAAAACAGATGTGAATAAAGAATTCCAAATGGGTGACAAGCCTACAAGTACTACA
G--GCAATGCTACA----
GCTCCAACCACTCTTACAGCAAGAGAGAATCCTGCTTACGGCCGACATATGCAGGATGCTGAGATGTTTACAA
ATGCCGCTTGCATGGCATTGAATATTTGGGATCGCTTTGATGTATTCTGTACACTAGGAGCCTCTAGCGGATAC
CTTAAAGGAAACTCTGCTTCTTTCAATTTAGTTGGATTGTTTGGAGATAATGAAAATCAAAGCACGGTCAA---
AACGAATTCTGTACCAAATATGAGCTTAGATCAATCTGTTGTTGAACTTTACACAGATACTGCCTTCTCTTGGAG
CGTGGGCGCTCGAGCAGCTTTGTGGGAGTGCGGATGTGCGACTTTAGGG

#FSW_6
CCTTGCAAGCTCTGCCTGTGGGGAATCCTGCTGAACCAAGCCTTATGATCGACGGAATTCTGTGGGAAGGTTT
CGGCGGAGATCCTTGCGATCCTTGCACCACTTGGTGTGACGCTATCAGCATGCGTATGGGTTACTATGGTGACT
TTGTTTTCGACCGTGTTTTGAAAACAGATGTGAATAAAGAATTCCAAATGGGTGACAAGCCTACAAGTACTACA
G--GCAATGCTACA----
GCTCCAACCACTCTTACAGCAAGAGAGAATCCTGCTTACGGCCGACATATGCAGGATGCTGAGATGTTTACAA
ATGCCGCTTGCATGGCATTGAATATTTGGGATCGCTTTGATGTATTCTGTACACTAGGAGCCTCTAGCGGATAC
CTTAAAGGAAACTCTGCTTCTTTCAATTTAGTTGGATTGTTTGGAGATAATGAAAATCAAAGCACGGTCAA---
AACGAATTCTGTACCAAATATGAGCTTAGATCAATCTGTTGTTGAACTTTACACAGATACTGCCTTCTCTTGGAG
CGTGGGCGCTCGAGCAGCTTTGTGGGAGTGCGGATGTGCGACTTTAGGG

#FSW_18
CCTTGCAAGCTCTGCCTGTGGGGAATCCTGCTGAACCAAGCCTTATGATCGACGGAATTCTGTGGGAAGGTTT
CGGCGGAGATCCTTGCGATCCTTGCACCACTTGGTGTGACGCTATCAGCATGCGTATGGGTTACTATGGTGACT
TTGTTTTCGACCGTGTTTTGAAAACAGATGTGAATAAAGAATTCCAAATGGGTGACAAGCCTACAAGTACTACA
G--GCAATGCTACA----
GCTCCAACCACTCTTACAGCAAGAGAGAATCCTGCTTACGGCCGACATATGCAGGATGCTGAGATGTTTACAA
ATGCCGCTTGCATGGCATTGAATATTTGGGATCGCTTTGATGTATTCTGTACACTAGGAGCCTCTAGCGGATAC
CTTAAAGGAAACTCTGCTTCTTTCAATTTAGTTGGATTGTTTGGAGATAATGAAAATCAAAGCACGGTCAA---
AACGAATTCTGTACCAAATATGAGCTTAGATCAATCTGTTGTTGAACTTTACACAGATACTGCCTTCTCTTGGAG
CGTGGGCGCTCGAGCAGCTTTGTGGGAGTGCGGATGTGCGACTTTAGGG

#FSW_23
CCTTGCAAGCTCTGCCTGTGGGGAATCCTGCTGAACCAAGCCTTATGATCGACGGAATTCTGTGGGAAGGTTT
CGGCGGAGATCCTTGCGATCCTTGCACCACTTGGTGTGACGCTATCAGCATGCGTATGGGTTACTATGGTGACT
TTGTTTTCGACCGTGTTTTGAAAACAGATGTGAATAAAGAATTCCAAATGGGTGACAAGCCTACAAGTACTACA
G--GCAATGCTACA----
GCTCCAACCACTCTTACAGCAAGAGAGAATCCTGCTTACGGCCGACATATGCAGGATGCTGAGATGTTTACAA
ATGCCGCTTGCATGGCATTGAATATTTGGGATCGCTTTGATGTATTCTGTACACTAGGAGCCTCTAGCGGATAC
CTTAAAGGAAACTCTGCTTCTTTCAATTTAGTTGGATTGTTTGGAGATAATGAAAATCAAAGCACGGTCAA---
AACGAATTCTGTACCAAATATGAGCTTAGATCAATCTGTTGTTGAACTTTACACAGATACTGCCTTCTCTTGGAG
CGTGGGCGCTCGAGCAGCTTTGTGGGAGTGCGGATGTGCGACTTTAGGG

#FSW_25_1
CCTTGCAAGCTCTGCCTGTGGGGAATCCTGCTGAACCAAGCCTTATGATCGACGGAATTCTGTGGGAAGGTTT
CGGCGGAGATCCTTGCGATCCTTGCACCACTTGGTGTGACGCTATCAGCATGCGTATGGGTTACTATGGTGACT
TTGTTTTCGACCGTGTTTTGAAAACAGATGTGAATAAAGAATTCCAAATGGGTGACAAGCCTACAAGTACTACA
G--GCAATGCTACA----
GCTCCAACCACTCTTACAGCAAGAGAGAATCCTGCTTACGGCCGACATATGCAGGATGCTGAGATGTTTACAA
ATGCCGCTTGCATGGCATTGAATATTTGGGATCGCTTTGATGTATTCTGTACACTAGGAGCCTCTAGCGGATAC
CTTAAAGGAAACTCTGCTTCTTTCAATTTAGTTGGATTGTTTGGAGATAATGAAAATCAAAGCACGGTCAA---
AACGAATTCTGTACCAAATATGAGCTTAGATCAATCTGTTGTTGAACTTTACACAGATACTGCCTTCTCTTGGAG
CGTGGGCGCTCGAGCAGCTTTGTGGGAGTGCGGATGTGCGACTTTAGGG

#FSW_25_2
CCTTGCAAGCTCTGCCTGTGGGGAATCCTGCTGAACCAAGCCTTATGATCGACGGAATTCTGTGGGAAGGTTT
CGGCGGAGATCCTTGCGATCCTTGCACCACTTGGTGTGACGCTATCAGCATGCGTATGGGTTACTATGGTGACT
TTGTTTTCGACCGTGTTTTGAAAACAGATGTGAATAAAGAATTCCAAATGGGTGACAAGCCTACAAGTACTACA
G--GCAATGCTACA----
GCTCCAACCACTCTTACAGCAAGAGAGAATCCTGCTTACGGCCGACATATGCAGGATGCTGAGATGTTTACAA
ATGCCGCTTGCATGGCATTGAATATTTGGGATCGCTTTGATGTATTCTGTACACTAGGAGCCTCTAGCGGATAC
CTTAAAGGAAACTCTGCTTCTTTCAATTTAGTTGGATTGTTTGGAGATAATGAAAATCAAAGCACGGTCAA---
AACGAATTCTGTACCAAATATGAGCTTAGATCAATCTGTTGTTGAACTTTACACAGATACTGCCTTCTCTTGGAG
CGTGGGCGCTCGAGCAGCTTTGTGGGAGTGCGGATGTGCGACTTTAGGG

#FSW_38
CCTTGCAAGCTCTGCCTGTGGGGAATCCTGCTGAACCAAGCCTTATGATCGACGGAATTCTGTGGGAAGGTTT
CGGCGGAGATCCTTGCGATCCTTGCACCACTTGGTGTGACGCTATCAGCATGCGTATGGGTTACTATGGTGACT
TTGTTTTCGACCGTGTTTTGAAAACAGATGTGAATAAAGAATTCCAAATGGGTGACAAGCCTACAAGTACTACA
G--GCAATGCTACA----
GCTCCAACCACTCTTACAGCAAGAGAGAATCCTGCTTACGGCCGACATATGCAGGATGCTGAGATGTTTACAA
ATGCCGCTTGCATGGCATTGAATATTTGGGATCGCTTTGATGTATTCTGTACACTAGGAGCCTCTAGCGGATAC
CTTAAAGGAAACTCTGCTTCTTTCAATTTAGTTGGATTGTTTGGAGATAATGAAAATCAAAGCACGGTCAA---
AACGAATTCTGTACCAAATATGAGCTTAGATCAATCTGTTGTTGAACTTTACACAGATACTGCCTTCTCTTGGAG
CGTGGGCGCTCGAGCAGCTTTGTGGGAGTGCGGATGTGCGACTTTAGGG

#FSW_58
CCTTGCAAGCTCTGCCTGTGGGGAATCCTGCTGAACCAAGCCTTATGATCGACGGAATTCTGTGGGAAGGTTT
CGGCGGAGATCCTTGCGATCCTTGCACCACTTGGTGTGACGCTATCAGCATGCGTATGGGTTACTATGGTGACT
TTGTTTTCGACCGTGTTTTGAAAACAGATGTGAATAAAGAATTCCAAATGGGTGACAAGCCTACAAGTACTACA
G--GCAATGCTACA----
GCTCCAACCACTCTTACAGCAAGAGAGAATCCTGCTTACGGCCGACATATGCAGGATGCTGAGATGTTTACAA
ATGCCGCTTGCATGGCATTGAATATTTGGGATCGCTTTGATGTATTCTGTACACTAGGAGCCTCTAGCGGATAC
CTTAAAGGAAACTCTGCTTCTTTCAATTTAGTTGGATTGTTTGGAGATAATGAAAATCAAAGCACGGTCAA---
AACGAATTCTGTACCAAATATGAGCTTAGATCAATCTGTTGTTGAACTTTACACAGATACTGCCTTCTCTTGGAG
CGTGGGCGCTCGAGCAGCTTTGTGGGAGTGCGGATGTGCGACTTTAGGG

#FSW_66
CCTTGCAAGCTCTGCCTGTGGGGAATCCTGCTGAACCAAGCCTTATGATCGACGGAATTCTGTGGGAAGGTTT
CGGCGGAGATCCTTGCGATCCTTGCACCACTTGGTGTGACGCTATCAGCATGCGTATGGGTTACTATGGTGACT
TTGTTTTCGACCGTGTTTTGAAAACAGATGTGAATAAAGAATTCCAAATGGGTGACAAGCCTACAAGTACTACA
G--GCAATGCTACA----
GCTCCAACCACTCTTACAGCAAGAGAGAATCCTGCTTACGGCCGACATATGCAGGATGCTGAGATGTTTACAA
ATGCCGCTTGCATGGCATTGAATATTTGGGATCGCTTTGATGTATTCTGTACACTAGGAGCCTCTAGCGGATAC
CTTAAAGGAAACTCTGCTTCTTTCAATTTAGTTGGATTGTTTGGAGATAATGAAAATCAAAGCACGGTCAA---
AACGAATTCTGTACCAAATATGAGCTTAGATCAATCTGTTGTTGAACTTTACACAGATACTGCCTTCTCTTGGAG
CGTGGGCGCTCGAGCAGCTTTGTGGGAGTGCGGATGTGCGACTTTAGGG

#FSW_71
CCTTGCAAGCTCTGCCTGTGGGGAATCCTGCTGAACCAAGCCTTATGATCGACGGAATTCTGTGGGAAGGTTT
CGGCGGAGATCCTTGCGATCCTTGCACCACTTGGTGTGACGCTATCAGCATGCGTATGGGTTACTATGGTGACT
TTGTTTTCGACCGTGTTTTGAAAACAGATGTGAATAAAGAATTCCAAATGGGTGACAAGCCTACAAGTACTACA
G--GCAATGCTACA----
GCTCCAACCACTCTTACAGCAAGAGAGAATCCTGCTTACGGCCGACATATGCAGGATGCTGAGATGTTTACAA
ATGCCGCTTGCATGGCATTGAATATTTGGGATCGCTTTGATGTATTCTGTACACTAGGAGCCTCTAGCGGATAC
CTTAAAGGAAACTCTGCTTCTTTCAATTTAGTTGGATTGTTTGGAGATAATGAAAATCAAAGCACGGTCAA---
AACGAATTCTGTACCAAATATGAGCTTAGATCAATCTGTTGTTGAACTTTACACAGATACTGCCTTCTCTTGGAG
CGTGGGCGCTCGAGCAGCTTTGTGGGAGTGCGGATGTGCGACTTTAGGG

#FSW_94
CCTTGCAAGCTCTGCCTGTGGGGAATCCTGCTGAACCAAGCCTTATGATCGACGGAATTCTGTGGGAAGGTTT
CGGCGGAGATCCTTGCGATCCTTGCACCACTTGGTGTGACGCTATCAGCATGCGTATGGGTTACTATGGTGACT
TTGTTTTCGACCGTGTTTTGAAAACAGATGTGAATAAAGAATTCCAAATGGGTGACAAGCCTACAAGTACTACA
G--GCAATGCTACA----
GCTCCAACCACTCTTACAGCAAGAGAGAATCCTGCTTACGGCCGACATATGCAGGATGCTGAGATGTTTACAA
ATGCCGCTTGCATGGCATTGAATATTTGGGATCGCTTTGATGTATTCTGTACACTAGGAGCCTCTAGCGGATAC
CTTAAAGGAAACTCTGCTTCTTTCAATTTAGTTGGATTGTTTGGAGATAATGAAAATCAAAGCACGGTCAA---
AACGAATTCTGTACCAAATATGAGCTTAGATCAATCTGTTGTTGAACTTTACACAGATACTGCCTTCTCTTGGAG
CGTGGGCGCTCGAGCAGCTTTGTGGGAGTGCGGATGTGCGACTTTAGGG

#FSW_100
CCTTGCAAGCTCTGCCTGTGGGGAATCCTGCTGAACCAAGCCTTATGATCGACGGAATTCTGTGGGAAGGTTT
CGGCGGAGATCCTTGCGATCCTTGCACCACTTGGTGTGACGCTATCAGCATGCGTATGGGTTACTATGGTGACT
TTGTTTTCGACCGTGTTTTGAAAACAGATGTGAATAAAGAATTCCAAATGGGTGACAAGCCTACAAGTACTACA
G--GCAATGCTACA----
GCTCCAACCACTCTTACAGCAAGAGAGAATCCTGCTTACGGCCGACATATGCAGGATGCTGAGATGTTTACAA
ATGCCGCTTGCATGGCATTGAATATTTGGGATCGCTTTGATGTATTCTGTACACTAGGAGCCTCTAGCGGATAC
CTTAAAGGAAACTCTGCTTCTTTCAATTTAGTTGGATTGTTTGGAGATAATGAAAATCAAAGCACGGTCAA---
AACGAATTCTGTACCAAATATGAGCTTAGATCAATCTGTTGTTGAACTTTACACAGATACTGCCTTCTCTTGGAG
CGTGGGCGCTCGAGCAGCTTTGTGGGAGTGCGGATGTGCGACTTTAGGG

#FSW_115
CCTTGCAAGCTCTGCCTGTGGGGAATCCTGCTGAACCAAGCCTTATGATCGACGGAATTCTGTGGGAAGGTTT
CGGCGGAGATCCTTGCGATCCTTGCACCACTTGGTGTGACGCTATCAGCATGCGTATGGGTTACTATGGTGACT
TTGTTTTCGACCGTGTTTTGAAAACAGATGTGAATAAAGAATTCCAAATGGGTGACAAGCCTACAAGTACTACA
G--GCAATGCTACA----
GCTCCAACCACTCTTACAGCAAGAGAGAATCCTGCTTACGGCCGACATATGCAGGATGCTGAGATGTTTACAA
ATGCCGCTTGCATGGCATTGAATATTTGGGATCGCTTTGATGTATTCTGTACACTAGGAGCCTCTAGCGGATAC
CTTAAAGGAAACTCTGCTTCTTTCAATTTAGTTGGATTGTTTGGAGATAATGAAAATCAAAGCACGGTCAA---
AACGAATTCTGTACCAAATATGAGCTTAGATCAATCTGTTGTTGAACTTTACACAGATACTGCCTTCTCTTGGAG
CGTGGGCGCTCGAGCAGCTTTGTGGGAGTGCGGATGTGCGACTTTAGGG

#FSW_119
CCTTGCAAGCTCTGCCTGTGGGGAATCCTGCTGAACCAAGCCTTATGATCGACGGAATTCTGTGGGAAGGTTT
CGGCGGAGATCCTTGCGATCCTTGCACCACTTGGTGTGACGCTATCAGCATGCGTATGGGTTACTATGGTGACT
TTGTTTTCGACCGTGTTTTGAAAACAGATGTGAATAAAGAATTCCAAATGGGTGACAAGCCTACAAGTACTACA
G--GCAATGCTACA----
GCTCCAACCACTCTTACAGCAAGAGAGAATCCTGCTTACGGCCGACATATGCAGGATGCTGAGATGTTTACAA
ATGCCGCTTGCATGGCATTGAATATTTGGGATCGCTTTGATGTATTCTGTACACTAGGAGCCTCTAGCGGATAC
CTTAAAGGAAACTCTGCTTCTTTCAATTTAGTTGGATTGTTTGGAGATAATGAAAATCAAAGCACGGTCAA---
AACGAATTCTGTACCAAATATGAGCTTAGATCAATCTGTTGTTGAACTTTACACAGATACTGCCTTCTCTTGGAG
CGTGGGCGCTCGAGCAGCTTTGTGGGAGTGCGGATGTGCGACTTTAGGG

#CP_62
CCTTGCAAGCTCTGCCTGTGGGGAATCCTGCTGAACCAAGCCTTATGATCGACGGAATTCTGTGGGAAGGTTT
CGGCGGAGATCCTTGCGATCCTTGCACCACTTGGTGTGACGCTATCAGCATGCGTATGGGTTACTACGGAGAC
TTTGTTTTCGACCGTGTTTTGAAAACTGATGTGAATAAAGAGTTTGAAATGGGCGAGGCTTTAGCCGGAGCTTC
TG--GGAATACGACCTC-
TACTCTTTCAAAATTGGTAGAACGAACGAACCCTGCATATGGCAAGCATATGCAAGACGCAGAGATGTTTACC
AATGCCGCTTGCATGGCATTGAATATTTGGGATCGTTTTGATGTATTCTGTACATTAGGAGCCACCAGTGGATA
TCTTAGAGGAAATTCAGCATCTTTCAACTTAGTTGGGTTATTCGGCGATAGTGAAAACGCCACGCAGCCTGC---
TGCAACAAGTATTCCTAACGTGCAGTTAAATCAGTCTGTGGTGGAACTGTATACAGATACTGCTTTTGCTTGGA
GTGTTGGAGCTCGTGCAGCTTTGTGGGAATGTGGATGCGCGACTTTAGGC

#CP_137
CCTTGCAAGCTCTGCCTGTGGGGAATCCTGCTGAACCAAGCCTTATGATCGACGGAATTCTGTGGGAAGGTTT
CGGCGGAGATCCTTGCGATCCTTGCACCACTTGGTGTGACGCTATCAGCATGCGTATGGGTTACTACGGAGAC
TTTGTTTTCGACCGTGTTTTGAAAACTGATGTGAATAAAGAGTTTGAAATGGGCGAGGCTTTAGCCGGAGCTTC
TG--GGAATACGACCTC-
TACTCTTTCAAAATTGGTAGAACGAACGAACCCTGCATATGGCAAGCATATGCAAGACGCAGAGATGTTTACC
AATGCCGCTTGCATGGCATTGAATATTTGGGATCGTTTTGATGTATTCTGTACATTAGGAGCCACCAGTGGATA
TCTTAGAGGAAATTCAGCATCTTTCAACTTAGTTGGGTTATTCGGCGATAGTGAAAACGCCACGCAGCCTGC---
TGCAACAAGTATTCCTAACGTGCAGTTAAATCAGTCTGTGGTGGAACTGTATACAGATACTGCTTTTGCTTGGA
GTGTTGGAGCTCGTGCAGCTTTGTGGGAATGTGGATGCGCGACTTTAGGC

#CP_140
CCTTGCAAGCTCTGCCTGTGGGGAATCCTGCTGAACCAAGCCTTATGATCGACGGAATTCTGTGGGAAGGTTT
CGGCGGAGATCCTTGCGATCCTTGCACCACTTGGTGTGACGCTATCAGCATGCGTATGGGTTACTACGGAGAC
TTTGTTTTCGACCGTGTTTTGAAAACTGATGTGAATAAAGAGTTTGAAATGGGCGAGGCTTTAGCCGGAGCTTC
TG--GGAATACGACCTC-
TACTCTTTCAAAATTGGTAGAACGAACGAACCCTGCATATGGCAAGCATATGCAAGACGCAGAGATGTTTACC
AATGCCGCTTGCATGGCATTGAATATTTGGGATCGTTTTGATGTATTCTGTACATTAGGAGCCACCAGTGGATA
TCTTAGAGGAAATTCAGCATCTTTCAACTTAGTTGGGTTATTCGGCGATAGTGAAAACGCCACGCAGCCTGC---
TGCAACAAGTATTCCTAACGTGCAGTTAAATCAGTCTGTGGTGGAACTGTATACAGATACTGCTTTTGCTTGGA
GTGTTGGAGCTCGTGCAGCTTTGTGGGAATGTGGATGCGCGACTTTAGGC

#CP_213_2
CCTTGCAAGCTCTGCCTGTGGGGAATCCTGCTGAACCAAGCCTTATGATCGACGGAATTCTGTGGGAAGGTTT
CGGCGGAGATCCTTGCGATCCTTGCACCACTTGGTGTGACGCTATCAGCATGCGTATGGGTTACTACGGAGAC
TTTGTTTTCGACCGTGTTTTGAAAACTGATGTGAATAAAGAGTTTGAAATGGGCGAGGCTTTAGCCGGAGCTTC
TG--GGAATACGACCTC-
TACTCTTTCAAAATTGGTAGAACGAACGAACCCTGCATATGGCAAGCATATGCAAGACGCAGAGATGTTTACC
AATGCCGCTTGCATGGCATTGAATATTTGGGATCGTTTTGATGTATTCTGTACATTAGGAGCCACCAGTGGATA
TCTTAGAGGAAATTCAGCATCTTTCAACTTAGTTGGGTTATTCGGCGATAGTGAAAACGCCACGCAGCCTGC---
TGCAACAAGTATTCCTAACGTGCAGTTAAATCAGTCTGTGGTGGAACTGTATACAGATACTGCTTTTGCTTGGA
GTGTTGGAGCTCGTGCAGCTTTGTGGGAATGTGGATGCGCGACTTTAGGC

#CP_217
CCTTGCAAGCTCTGCCTGTGGGGAATCCTGCTGAACCAAGCCTTATGATCGACGGAATTCTGTGGGAAGGTTT
CGGCGGAGATCCTTGCGATCCTTGCACCACTTGGTGTGACGCTATCAGCATGCGTATGGGTTACTACGGAGAC
TTTGTTTTCGACCGTGTTTTGAAAACTGATGTGAATAAAGAGTTTGAAATGGGCGAGGCTTTAGCCGGAGCTTC
TG--GGAATACGACCTC-
TACTCTTTCAAAATTGGTAGAACGAACGAACCCTGCATATGGCAAGCATATGCAAGACGCAGAGATGTTTACC
AATGCCGCTTGCATGGCATTGAATATTTGGGATCGTTTTGATGTATTCTGTACATTAGGAGCCACCAGTGGATA
TCTTAGAGGAAATTCAGCATCTTTCAACTTAGTTGGGTTATTCGGCGATAGTGAAAACGCCACGCAGCCTGC---
TGCAACAAGTATTCCTAACGTGCAGTTAAATCAGTCTGTGGTGGAACTGTATACAGATACTGCTTTTGCTTGGA
GTGTTGGAGCTCGTGCAGCTTTGTGGGAATGTGGATGCGCGACTTTAGGC

#CP_232
CCTTGCAAGCTCTGCCTGTGGGGAATCCTGCTGAACCAAGCCTTATGATCGACGGAATTCTGTGGGAAGGTTT
CGGCGGAGATCCTTGCGATCCTTGCACCACTTGGTGTGACGCTATCAGCATGCGTATGGGTTACTACGGAGAC
TTTGTTTTCGACCGTGTTTTGAAAACTGATGTGAATAAAGAGTTTGAAATGGGCGAGGCTTTAGCCGGAGCTTC
TG--GGAATACGACCTC-
TACTCTTTCAAAATTGGTAGAACGAACGAACCCTGCATATGGCAAGCATATGCAAGACGCAGAGATGTTTACC
AATGCCGCTTGCATGGCATTGAATATTTGGGATCGTTTTGATGTATTCTGTACATTAGGAGCCACCAGTGGATA
TCTTAGAGGAAATTCAGCATCTTTCAACTTAGTTGGGTTATTCGGCGATAGTGAAAACGCCACGCAGCCTGC---
TGCAACAAGTATTCCTAACGTGCAGTTAAATCAGTCTGTGGTGGAACTGTATACAGATACTGCTTTTGCTTGGA
GTGTTGGAGCTCGTGCAGCTTTGTGGGAATGTGGATGCGCGACTTTAGGC

#CP_258
CCTTGCAAGCTCTGCCTGTGGGGAATCCTGCTGAACCAAGCCTTATGATCGACGGAATTCTGTGGGAAGGTTT
CGGCGGAGATCCTTGCGATCCTTGCACCACTTGGTGTGACGCTATCAGCATGCGTATGGGTTACTACGGAGAC
TTTGTTTTCGACCGTGTTTTGAAAACTGATGTGAATAAAGAGTTTGAAATGGGCGAGGCTTTAGCCGGAGCTTC
TG--GGAATACGACCTC-
TACTCTTTCAAAATTGGTAGAACGAACGAACCCTGCATATGGCAAGCATATGCAAGACGCAGAGATGTTTACC
AATGCCGCTTGCATGGCATTGAATATTTGGGATCGTTTTGATGTATTCTGTACATTAGGAGCCACCAGTGGATA
TCTTAGAGGAAATTCAGCATCTTTCAACTTAGTTGGGTTATTCGGCGATAGTGAAAACGCCACGCAGCCTGC---
TGCAACAAGTATTCCTAACGTGCAGTTAAATCAGTCTGTGGTGGAACTGTATACAGATACTGCTTTTGCTTGGA
GTGTTGGAGCTCGTGCAGCTTTGTGGGAATGTGGATGCGCGACTTTAGGC

#CP_319
CCTTGCAAGCTCTGCCTGTGGGGAATCCTGCTGAACCAAGCCTTATGATCGACGGAATTCTGTGGGAAGGTTT
CGGCGGAGATCCTTGCGATCCTTGCACCACTTGGTGTGACGCTATCAGCATGCGTATGGGTTACTACGGAGAC
TTTGTTTTCGACCGTGTTTTGAAAACTGATGTGAATAAAGAGTTTGAAATGGGCGAGGCTTTAGCCGGAGCTTC
TG--GGAATACGACCTC-
TACTCTTTCAAAATTGGTAGAACGAACGAACCCTGCATATGGCAAGCATATGCAAGACGCAGAGATGTTTACC
AATGCCGCTTGCATGGCATTGAATATTTGGGATCGTTTTGATGTATTCTGTACATTAGGAGCCACCAGTGGATA
TCTTAGAGGAAATTCAGCATCTTTCAACTTAGTTGGGTTATTCGGCGATAGTGAAAACGCCACGCAGCCTGC---
TGCAACAAGTATTCCTAACGTGCAGTTAAATCAGTCTGTGGTGGAACTGTATACAGATACTGCTTTTGCTTGGA
GTGTTGGAGCTCGTGCAGCTTTGTGGGAATGTGGATGCGCGACTTTAGGC

#CP_384
CCTTGCAAGCTCTGCCTGTGGGGAATCCTGCTGAACCAAGCCTTATGATCGACGGAATTCTGTGGGAAGGTTT
CGGCGGAGATCCTTGCGATCCTTGCACCACTTGGTGTGACGCTATCAGCATGCGTATGGGTTACTACGGAGAC
TTTGTTTTCGACCGTGTTTTGAAAACTGATGTGAATAAAGAGTTTGAAATGGGCGAGGCTTTAGCCGGAGCTTC
TG--GGAATACGACCTC-
TACTCTTTCAAAATTGGTAGAACGAACGAACCCTGCATATGGCAAGCATATGCAAGACGCAGAGATGTTTACC
AATGCCGCTTGCATGGCATTGAATATTTGGGATCGTTTTGATGTATTCTGTACATTAGGAGCCACCAGTGGATA
TCTTAGAGGAAATTCAGCATCTTTCAACTTAGTTGGGTTATTCGGCGATAGTGAAAACGCCACGCAGCCTGC---
TGCAACAAGTATTCCTAACGTGCAGTTAAATCAGTCTGTGGTGGAACTGTATACAGATACTGCTTTTGCTTGGA
GTGTTGGAGCTCGTGCAGCTTTGTGGGAATGTGGATGCGCGACTTTAGGC

#FSW_82
CCTTGCAAGCTCTGCCTGTGGGGAATCCTGCTGAACCAAGCCTTATGATCGACGGAATTCTGTGGGAAGGTTT
CGGCGGAGATCCTTGCGATCCTTGCACCACTTGGTGTGACGCTATCAGCATGCGTATGGGTTACTACGGAGAC
TTTGTTTTCGACCGTGTTTTGAAAACAGATGTGAATAAAGAGTTTGAAATGGGCGAGGCTTTAGCCGGAGCTT
CTG--GGAATACGACCTC-
TACTCTTTCAAAATTGGTAGAACGAACGAACCCTGCATATGGCAAGCATATGCAAGACGCAGAGATGTTTACC
AATGCCGCTTGCATGGCATTGAATATTTGGGATCGTTTTGATGTATTCTGTACATTAGGAGCCACCAGTGGATA
TCTTAGAGGAAATTCAGCATCTTTCAACTTAGTTGGGTTATTCGGCGATAGTGAAAACGCCACGCAGCCTGC---
TGCAACAAGTATTCCTAACGTGCAGTTAAATCAGTCTGTGGTGGAACTGTATACAGATACTGCTTTTGCTTGGA
GTGTTGGAGCTCGTGCAGCTTTGTGGGAATGTGGATGCGCGACTTTAGGC

#CP_247
CCTTGCAAGCTCTGCCTGTGGGGAATCCTGCTGAACCAAGCCTTATGATCGACGGAATTCTGTGGGAAGGTTT
CGGCGGAGATCCTTGCGATCCTTGCACCACTTGGTGTGACGCTATCAGCATGCGCGTTGGTTACTACGGAGAC
TTTGTTTTCGACCGTGTTTTGAAAACTGATGTGAATAAAGAATTTCAGATGGGAGCGGCGCCTACTACCAGCGA
TGTAGAAGGCTTACAAAACGATCCAACAACAAATGTTGCTCGTCCAAATCCCGCTTATGGCAAACACATGCAA
GATGCTGAAATGTTTACGAACGCTGCTTACATGGCATTAAATATCTGGGATCGTTTTGATGTATTTTGTACATTG
GGAGCAACTACCGGTTATTTAAGAGGAAACTCCGCTTCCTTCAACTTAGTTGGATTATTCGGAACAAAAACACA
ATATTCTAAGTTTAATACAGCGAATCTTGTTCCTAACACTGCTTTGGATCGAGCTGTGGTTGAGCTTTATACAGA
CACCACCTTTGCTTGGAGCGTAGGTGCTCGTGCAGCTCTCTGGGAATGTGGGTGTGCAACGTTAGGA

#CP_446_1
CCTTGCAAGCTCTGCCTGTGGGGAATCCTGCTGAACCAAGCCTTATGATCGACGGAATTCTGTGGGAAGGTTT
CGGCGGAGATCCTTGCGATCCTTGCACCACTTGGTGTGACGCTATCAGCATGCGCGTTGGTTACTACGGAGAC
TTTGTTTTCGACCGTGTTTTGAAAACTGATGTGAATAAAGAATTTCAGATGGGAGCGGCGCCTACTACCAGCGA
TGTAGAAGGCTTACAAAACGATCCAACAACAAATGTTGCTCGTCCAAATCCCGCTTATGGCAAACACATGCAA
GATGCTGAAATGTTTACGAACGCTGCTTACATGGCATTAAATATCTGGGATCGTTTTGATGTATTTTGTACATTG
GGAGCAACTACCGGTTATTTAAGAGGAAACTCCGCTTCCTTCAACTTAGTTGGATTATTCGGAACAAAAACACA
ATATTCTAAGTTTAATACAGCGAATCTTGTTCCTAACACTGCTTTGGATCGAGCTGTGGTTGAGCTTTATACAGA
CACCACCTTTGCTTGGAGCGTAGGTGCTCGTGCAGCTCTCTGGGAATGTGGGTGTGCAACGTTAGGA

#CP_446_2
CCTTGCAAGCTCTGCCTGTGGGGAATCCTGCTGAACCAAGCCTTATGATCGACGGAATTCTGTGGGAAGGTTT
CGGCGGAGATCCTTGCGATCCTTGCACCACTTGGTGTGACGCTATCAGCATGCGCGTTGGTTACTACGGAGAC
TTTGTTTTCGACCGTGTTTTGAAAACTGATGTGAATAAAGAATTTCAGATGGGAGCGGCGCCTACTACCAGCGA

TGTAGAAGGCTTACAAAACGATCCAACAACAAATGTTGCTCGTCCAAATCCCGCTTATGGCAAACACATGCAA
GATGCTGAAATGTTTACGAACGCTGCTTACATGGCATTAAATATCTGGGATCGTTTTGATGTATTTTGTACATTG
GGAGCAACTACCGGTTATTTAAGAGGAAACTCCGCTTCCTTCAACTTAGTTGGATTATTCGGAACAAAAACACA
ATATTCTAAGTTTAATACAGCGAATCTTGTTCCTAACACTGCTTTGGATCGAGCTGTGGTTGAGCTTTATACAGA
CACCACCTTTGCTTGGAGCGTAGGTGCTCGTGCAGCTCTCTGGGAATGTGGGTGTGCAACGTTAGGA

#FSW_10
CCTTGCAAGCTCTGCCTGTGGGGAATCCTGCTGAACCAAGCCTTATGATCGACGGAATTCTGTGGGAAGGTTT
CGGCGGAGATCCTTGCGATCCTTGCACCACTTGGTGTGACGCTATCAGCATGCGCGTTGGTTACTACGGAGAC
TTTGTTTTCGACCGTGTTTTGAAAACTGATGTGAATAAAGAATTTCAGATGGGAGCGGCGCCTACTACCAGCGA
TGTAGAAGGCTTACAAAACGATCCAACAACAAATGTTGCTCGTCCAAATCCCGCTTATGGCAAACACATGCAA
GATGCTGAAATGTTTACGAACGCTGCTTACATGGCATTAAATATCTGGGATCGTTTTGATGTATTTTGTACATTG
GGAGCAACTACCGGTTATTTAAGAGGAAACTCCGCTTCCTTCAACTTAGTTGGATTATTCGGAACAAAAACACA
ATATTCTAAGTTTAATACAGCGAATCTTGTTCCTAACACTGCTTTGGATCGAGCTGTGGTTGAGCTTTATACAGA
CACCACCTTTGCTTGGAGCGTAGGTGCTCGTGCAGCTCTCTGGGAATGTGGGTGTGCAACGTTAGGA

#CP_121
CCTTGCAAGCTCTGCCTGTGGGGAATCCTGCTGAACCAAGCCTTATGATCGACGGAATTCTGTGGGAAGGTTT
CGGCGGAGATCCTTGCGATCCTTGCACCACTTGGTGTGACGCTATCAGCATGCGTATGGGTTACTACGGAGAC
TTTGTTTTCGACCGTGTTTTGAAAACAGATGTGAATAAAGAATTTCAGATGGGAGCGGCGCCTACTACCAAGG
ATGTAGCAGGCTTAGAAAACGATCCAACAACAAATGTTGCTCGTCCAAATCCCGCTTATGGCAAACACATGCA
AGATGCTGAAATGTTTACGAACGCTGCTTACATGGCATTAAATATCTGGGATCGTTTTGATGTATTTTGTACATT
GGGAGCAACTACCGGTTATTTAAAAGGAAACTCCGCTTCCTTCAACTTAGTTGGATTATTCGGAACAAAAACAC
AATCTTCTAACTTTAATACAGCGAAGCTTGTTCCTAACGCTGCTTTGAATCAAGCTGTGGTTGAGCTTTATACAG
ACACTACCTTTGCTTGGAGCGTAGGTGCTCGTGCAGCTCTCTGGGAATGTGGGTGTGCAACGTTAGGA

#CP_15
CCTTGCAAGCTCTGCCTGTGGGGAATCCTGCTGAACCAAGCCTTATGATCGACGGAATTCTGTGGGAAGGTTT
CGGCGGAGATCCTTGCGATCCTTGCACCACTTGGTGTGACGCTATCAGCATGCGTATGGGTTACTATGGTGACT
TTGTTTTCGACCGTGTTTTGAAAACAGATGTGAATAAAGAGTTTGAAATGGGCGAGGCTTTAGCCGGAGCTTC
TG--GGAATACGACCTC-
TACTCTTTCAAAATTGGTAGAACGAACGAACCCTGCATATGGCAAGCATATGCAAGACGCAGAGATGTTTACC
AATGCCGCTTGCATGACATTGAATATTTGGGATCGTTTTGATGTATTCTGTACATTAGGAGCCACCAGTGGATA
TCTTAAAGGAAATTCAGCATCTTTCAACTTAGTTGGGTTATTCGGCGATGGTGTAAACGCCACGAAACCTGC---
TGCAGATAGTATTCCTAACGTGCAGTTAAATCAGTCTGTGGTGGAACTGTATACAGATACTACTTTTGCTTGGA
GTGTTGGAGCTCGTGCAGCTTTGTGGGAATGTGGATGTGCAACTTTAGGA

#CP_39
CCTTGCAAGCTCTGCCTGTGGGGAATCCTGCTGAACCAAGCCTTATGATCGACGGAATTCTGTGGGAAGGTTT
CGGCGGAGATCCTTGCGATCCTTGCACCACTTGGTGTGACGCTATCAGCATGCGTATGGGTTACTATGGTGACT
TTGTTTTCGACCGTGTTTTGAAAACAGATGTGAATAAAGAGTTTGAAATGGGCGAGGCTTTAGCCGGAGCTTC
TG--GGAATACGACCTC-
TACTCTTTCAAAATTGGTAGAACGAACGAACCCTGCATATGGCAAGCATATGCAAGACGCAGAGATGTTTACC
AATGCCGCTTGCATGACATTGAATATTTGGGATCGTTTTGATGTATTCTGTACATTAGGAGCCACCAGTGGATA
TCTTAAAGGAAATTCAGCATCTTTCAACTTAGTTGGGTTATTCGGCGATGGTGTAAACGCCACGAAACCTGC---

TGCAGATAGTATTCCTAACGTGCAGTTAAATCAGTCTGTGGTGGAACTGTATACAGATACTACTTTTGCTTGGA
GTGTTGGAGCTCGTGCAGCTTTGTGGGAATGTGGATGTGCAACTTTAGGA

#CP_81
CCTTGCAAGCTCTGCCTGTGGGGAATCCTGCTGAACCAAGCCTTATGATCGACGGAATTCTGTGGGAAGGTTT
CGGCGGAGATCCTTGCGATCCTTGCACCACTTGGTGTGACGCTATCAGCATGCGTATGGGTTACTATGGTGACT
TTGTTTTCGACCGTGTTTTGAAAACAGATGTGAATAAAGAGTTTGAAATGGGCGAGGCTTTAGCCGGAGCTTC
TG--GGAATACGACCTC-
TACTCTTTCAAAATTGGTAGAACGAACGAACCCTGCATATGGCAAGCATATGCAAGACGCAGAGATGTTTACC
AATGCCGCTTGCATGACATTGAATATTTGGGATCGTTTTGATGTATTCTGTACATTAGGAGCCACCAGTGGATA
TCTTAAAGGAAATTCAGCATCTTTCAACTTAGTTGGGTTATTCGGCGATGGTGTAAACGCCACGAAACCTGC---
TGCAGATAGTATTCCTAACGTGCAGTTAAATCAGTCTGTGGTGGAACTGTATACAGATACTACTTTTGCTTGGA
GTGTTGGAGCTCGTGCAGCTTTGTGGGAATGTGGATGTGCAACTTTAGGA

#CP_120_2
CCTTGCAAGCTCTGCCTGTGGGGAATCCTGCTGAACCAAGCCTTATGATCGACGGAATTCTGTGGGAAGGTTT
CGGCGGAGATCCTTGCGATCCTTGCACCACTTGGTGTGACGCTATCAGCATGCGTATGGGTTACTATGGTGACT
TTGTTTTCGACCGTGTTTTGAAAACAGATGTGAATAAAGAGTTTGAAATGGGCGAGGCTTTAGCCGGAGCTTC
TG--GGAATACGACCTC-
TACTCTTTCAAAATTGGTAGAACGAACGAACCCTGCATATGGCAAGCATATGCAAGACGCAGAGATGTTTACC
AATGCCGCTTGCATGACATTGAATATTTGGGATCGTTTTGATGTATTCTGTACATTAGGAGCCACCAGTGGATA
TCTTAAAGGAAATTCAGCATCTTTCAACTTAGTTGGGTTATTCGGCGATGGTGTAAACGCCACGAAACCTGC---
TGCAGATAGTATTCCTAACGTGCAGTTAAATCAGTCTGTGGTGGAACTGTATACAGATACTACTTTTGCTTGGA
GTGTTGGAGCTCGTGCAGCTTTGTGGGAATGTGGATGTGCAACTTTAGGA

#CP_298
CCTTGCAAGCTCTGCCTGTGGGGAATCCTGCTGAACCAAGCCTTATGATCGACGGAATTCTGTGGGAAGGTTT
CGGCGGAGATCCTTGCGATCCTTGCACCACTTGGTGTGACGCTATCAGCATGCGTATGGGTTACTATGGTGACT
TTGTTTTCGACCGTGTTTTGAAAACAGATGTGAATAAAGAGTTTGAAATGGGCGAGGCTTTAGCCGGAGCTTC
TG--GGAATACGACCTC-
TACTCTTTCAAAATTGGTAGAACGAACGAACCCTGCATATGGCAAGCATATGCAAGACGCAGAGATGTTTACC
AATGCCGCTTGCATGACATTGAATATTTGGGATCGTTTTGATGTATTCTGTACATTAGGAGCCACCAGTGGATA
TCTTAAAGGAAATTCAGCATCTTTCAACTTAGTTGGGTTATTCGGCGATGGTGTAAACGCCACGAAACCTGC---
TGCAGATAGTATTCCTAACGTGCAGTTAAATCAGTCTGTGGTGGAACTGTATACAGATACTACTTTTGCTTGGA
GTGTTGGAGCTCGTGCAGCTTTGTGGGAATGTGGATGTGCAACTTTAGGA

#CP_302
CCTTGCAAGCTCTGCCTGTGGGGAATCCTGCTGAACCAAGCCTTATGATCGACGGAATTCTGTGGGAAGGTTT
CGGCGGAGATCCTTGCGATCCTTGCACCACTTGGTGTGACGCTATCAGCATGCGTATGGGTTACTATGGTGACT
TTGTTTTCGACCGTGTTTTGAAAACAGATGTGAATAAAGAGTTTGAAATGGGCGAGGCTTTAGCCGGAGCTTC
TG--GGAATACGACCTC-
TACTCTTTCAAAATTGGTAGAACGAACGAACCCTGCATATGGCAAGCATATGCAAGACGCAGAGATGTTTACC
AATGCCGCTTGCATGACATTGAATATTTGGGATCGTTTTGATGTATTCTGTACATTAGGAGCCACCAGTGGATA
TCTTAAAGGAAATTCAGCATCTTTCAACTTAGTTGGGTTATTCGGCGATGGTGTAAACGCCACGAAACCTGC---
TGCAGATAGTATTCCTAACGTGCAGTTAAATCAGTCTGTGGTGGAACTGTATACAGATACTACTTTTGCTTGGA
GTGTTGGAGCTCGTGCAGCTTTGTGGGAATGTGGATGTGCAACTTTAGGA

#FSW_92
CCTTGCAAGCTCTGCCTGTGGGGAATCCTGCTGAACCAAGCCTTATGATCGACGGAATTCTGTGGGAAGGTTT
CGGCGGAGATCCTTGCGATCCTTGCACCACTTGGTGTGACGCTATCAGCATGCGTATGGGTTACTATGGTGACT
TTGTTTTCGACCGTGTTTTGAAAACAGATGTGAATAAAGAGTTTGAAATGGGCGAGGCTTTAGCCGGAGCTTC
TG--GGAATACGACCTC-
TACTCTTTCAAAATTGGTAGAACGAACGAACCCTGCATATGGCAAGCATATGCAAGACGCAGAGATGTTTACC
AATGCCGCTTGCATGACATTGAATATTTGGGATCGTTTTGATGTATTCTGTACATTAGGAGCCACCAGTGGATA
TCTTAAAGGAAATTCAGCATCTTTCAACTTAGTTGGGTTATTCGGCGATGGTGTAAACGCCACGAAACCTGC---
TGCAGATAGTATTCCTAACGTGCAGTTAAATCAGTCTGTGGTGGAACTGTATACAGATACTACTTTTGCTTGGA
GTGTTGGAGCTCGTGCAGCTTTGTGGGAATGTGGATGTGCAACTTTAGGA

#CP_58_2
CCTTGCAAGCTCTGCCTGTGGGGAATCCTGCTGAACCAAGCCTTATGATCGACGGAATTCTGTGGGAAGGTTTT
GGCGGAGATCCTTGCGATCCTTGCGCCACTTGGTGTGACGCTATCAGCATGCGTGTTGGTTACTACGGAGACT
TTGTTTTCGACCGTGTTTTGAAAACTGATGTGAATAAAGAATTTCAGATGGGAGCGGCGCCTACTACCAGCGAT
GCAGCAGACTTACAAAACGATCCAAAAACAAATGTTGCTCGTCCAAATCCCGCTTATGGCAAACACATGCAAG
ATGCTGAAATGTTTACGAACGCTGCTTACATGGCATTAAATATCTGGGATCGTTTTGATGTATTTTGTACATTGG
GAGCAACTACCGGTTATTTAAAAGGAAACTCCGCTTCCTTCAACTTAGTTGGATTATTCGGAACAAAAACAAAA
TCTTCTGATTTTAATACAGCGAAGCTTGTTCCTAACATTGCTTTGAATCGAGCTGTGGTTGAGCTTTATACAGAC
ACTACCTTTGCTTGGAGCGTAGGTGCTCGTGCAGCTCTCTGGGAATGTGGGTGTGCAACGTTAGGA

#CP_138
CCTTGCAAGCTCTGCCTGTGGGGAATCCTGCTGAACCAAGCCTTATGATCGACGGAATTCTGTGGGAAGGTTTT
GGCGGAGATCCTTGCGATCCTTGCGCCACTTGGTGTGACGCTATCAGCATGCGTGTTGGTTACTACGGAGACT
TTGTTTTCGACCGTGTTTTGAAAACTGATGTGAATAAAGAATTTCAGATGGGAGCGGCGCCTACTACCAGCGAT
GCAGCAGACTTACAAAACGATCCAAAAACAAATGTTGCTCGTCCAAATCCCGCTTATGGCAAACACATGCAAG
ATGCTGAAATGTTTACGAACGCTGCTTACATGGCATTAAATATCTGGGATCGTTTTGATGTATTTTGTACATTGG
GAGCAACTACCGGTTATTTAAAAGGAAACTCCGCTTCCTTCAACTTAGTTGGATTATTCGGAACAAAAACAAAA
TCTTCTGATTTTAATACAGCGAAGCTTGTTCCTAACATTGCTTTGAATCGAGCTGTGGTTGAGCTTTATACAGAC
ACTACCTTTGCTTGGAGCGTAGGTGCTCGTGCAGCTCTCTGGGAATGTGGGTGTGCAACGTTAGGA

#CP_208
CCTTGCAAGCTCTGCCTGTGGGGAATCCTGCTGAACCAAGCCTTATGATCGACGGAATTCTGTGGGAAGGTTTT
GGCGGAGATCCTTGCGATCCTTGCGCCACTTGGTGTGACGCTATCAGCATGCGTGTTGGTTACTACGGAGACT
TTGTTTTCGACCGTGTTTTGAAAACTGATGTGAATAAAGAATTTCAGATGGGAGCGGCGCCTACTACCAGCGAT
GCAGCAGACTTACAAAACGATCCAAAAACAAATGTTGCTCGTCCAAATCCCGCTTATGGCAAACACATGCAAG
ATGCTGAAATGTTTACGAACGCTGCTTACATGGCATTAAATATCTGGGATCGTTTTGATGTATTTTGTACATTGG
GAGCAACTACCGGTTATTTAAAAGGAAACTCCGCTTCCTTCAACTTAGTTGGATTATTCGGAACAAAAACAAAA
TCTTCTGATTTTAATACAGCGAAGCTTGTTCCTAACATTGCTTTGAATCGAGCTGTGGTTGAGCTTTATACAGAC
ACTACCTTTGCTTGGAGCGTAGGTGCTCGTGCAGCTCTCTGGGAATGTGGGTGTGCAACGTTAGGA

#CP_241
CCTTGCAAGCTCTGCCTGTGGGGAATCCTGCTGAACCAAGCCTTATGATCGACGGAATTCTGTGGGAAGGTTTT
GGCGGAGATCCTTGCGATCCTTGCGCCACTTGGTGTGACGCTATCAGCATGCGTGTTGGTTACTACGGAGACT
TTGTTTTCGACCGTGTTTTGAAAACTGATGTGAATAAAGAATTTCAGATGGGAGCGGCGCCTACTACCAGCGAT
GCAGCAGACTTACAAAACGATCCAAAAACAAATGTTGCTCGTCCAAATCCCGCTTATGGCAAACACATGCAAG

ATGCTGAAATGTTTACGAACGCTGCTTACATGGCATTAAATATCTGGGATCGTTTTGATGTATTTTGTACATTGG
GAGCAACTACCGGTTATTTAAAAGGAAACTCCGCTTCCTTCAACTTAGTTGGATTATTCGGAACAAAAACAAAA
TCTTCTGATTTTAATACAGCGAAGCTTGTTCCTAACATTGCTTTGAATCGAGCTGTGGTTGAGCTTTATACAGAC
ACTACCTTTGCTTGGAGCGTAGGTGCTCGTGCAGCTCTCTGGGAATGTGGGTGTGCAACGTTAGGA

#CP_266
CCTTGCAAGCTCTGCCTGTGGGGAATCCTGCTGAACCAAGCCTTATGATCGACGGAATTCTGTGGGAAGGTTTT
GGCGGAGATCCTTGCGATCCTTGCGCCACTTGGTGTGACGCTATCAGCATGCGTGTTGGTTACTACGGAGACT
TTGTTTTCGACCGTGTTTTGAAAACTGATGTGAATAAAGAATTTCAGATGGGAGCGGCGCCTACTACCAGCGAT
GCAGCAGACTTACAAAACGATCCAAAAACAAATGTTGCTCGTCCAAATCCCGCTTATGGCAAACACATGCAAG
ATGCTGAAATGTTTACGAACGCTGCTTACATGGCATTAAATATCTGGGATCGTTTTGATGTATTTTGTACATTGG
GAGCAACTACCGGTTATTTAAAAGGAAACTCCGCTTCCTTCAACTTAGTTGGATTATTCGGAACAAAAACAAAA
TCTTCTGATTTTAATACAGCGAAGCTTGTTCCTAACATTGCTTTGAATCGAGCTGTGGTTGAGCTTTATACAGAC
ACTACCTTTGCTTGGAGCGTAGGTGCTCGTGCAGCTCTCTGGGAATGTGGGTGTGCAACGTTAGGA

#CP_303
CCTTGCAAGCTCTGCCTGTGGGGAATCCTGCTGAACCAAGCCTTATGATCGACGGAATTCTGTGGGAAGGTTTT
GGCGGAGATCCTTGCGATCCTTGCGCCACTTGGTGTGACGCTATCAGCATGCGTGTTGGTTACTACGGAGACT
TTGTTTTCGACCGTGTTTTGAAAACTGATGTGAATAAAGAATTTCAGATGGGAGCGGCGCCTACTACCAGCGAT
GCAGCAGACTTACAAAACGATCCAAAAACAAATGTTGCTCGTCCAAATCCCGCTTATGGCAAACACATGCAAG
ATGCTGAAATGTTTACGAACGCTGCTTACATGGCATTAAATATCTGGGATCGTTTTGATGTATTTTGTACATTGG
GAGCAACTACCGGTTATTTAAAAGGAAACTCCGCTTCCTTCAACTTAGTTGGATTATTCGGAACAAAAACAAAA
TCTTCTGATTTTAATACAGCGAAGCTTGTTCCTAACATTGCTTTGAATCGAGCTGTGGTTGAGCTTTATACAGAC
ACTACCTTTGCTTGGAGCGTAGGTGCTCGTGCAGCTCTCTGGGAATGTGGGTGTGCAACGTTAGGA

#FSW_16
CCTTGCAAGCTCTGCCTGTGGGGAATCCTGCTGAACCAAGCCTTATGATCGACGGAATTCTGTGGGAAGGTTTT
GGCGGAGATCCTTGCGATCCTTGCGCCACTTGGTGTGACGCTATCAGCATGCGTGTTGGTTACTACGGAGACT
TTGTTTTCGACCGTGTTTTGAAAACTGATGTGAATAAAGAATTTCAGATGGGAGCGGCGCCTACTACCAGCGAT
GCAGCAGACTTACAAAACGATCCAAAAACAAATGTTGCTCGTCCAAATCCCGCTTATGGCAAACACATGCAAG
ATGCTGAAATGTTTACGAACGCTGCTTACATGGCATTAAATATCTGGGATCGTTTTGATGTATTTTGTACATTGG
GAGCAACTACCGGTTATTTAAAAGGAAACTCCGCTTCCTTCAACTTAGTTGGATTATTCGGAACAAAAACAAAA
TCTTCTGATTTTAATACAGCGAAGCTTGTTCCTAACATTGCTTTGAATCGAGCTGTGGTTGAGCTTTATACAGAC
ACTACCTTTGCTTGGAGCGTAGGTGCTCGTGCAGCTCTCTGGGAATGTGGGTGTGCAACGTTAGGA

#FSW_21
CCTTGCAAGCTCTGCCTGTGGGGAATCCTGCTGAACCAAGCCTTATGATCGACGGAATTCTGTGGGAAGGTTTT
GGCGGAGATCCTTGCGATCCTTGCGCCACTTGGTGTGACGCTATCAGCATGCGTGTTGGTTACTACGGAGACT
TTGTTTTCGACCGTGTTTTGAAAACTGATGTGAATAAAGAATTTCAGATGGGAGCGGCGCCTACTACCAGCGAT
GCAGCAGACTTACAAAACGATCCAAAAACAAATGTTGCTCGTCCAAATCCCGCTTATGGCAAACACATGCAAG
ATGCTGAAATGTTTACGAACGCTGCTTACATGGCATTAAATATCTGGGATCGTTTTGATGTATTTTGTACATTGG
GAGCAACTACCGGTTATTTAAAAGGAAACTCCGCTTCCTTCAACTTAGTTGGATTATTCGGAACAAAAACAAAA
TCTTCTGATTTTAATACAGCGAAGCTTGTTCCTAACATTGCTTTGAATCGAGCTGTGGTTGAGCTTTATACAGAC
ACTACCTTTGCTTGGAGCGTAGGTGCTCGTGCAGCTCTCTGGGAATGTGGGTGTGCAACGTTAGGA

#FSW_60
CCTTGCAAGCTCTGCCTGTGGGGAATCCTGCTGAACCAAGCCTTATGATCGACGGAATTCTGTGGGAAGGTTTT
GGCGGAGATCCTTGCGATCCTTGCGCCACTTGGTGTGACGCTATCAGCATGCGTGTTGGTTACTACGGAGACT
TTGTTTTCGACCGTGTTTTGAAAACTGATGTGAATAAAGAATTTCAGATGGGAGCGGCGCCTACTACCAGCGAT
GCAGCAGACTTACAAAACGATCCAAAAACAAATGTTGCTCGTCCAAATCCCGCTTATGGCAAACACATGCAAG
ATGCTGAAATGTTTACGAACGCTGCTTACATGGCATTAAATATCTGGGATCGTTTTGATGTATTTTGTACATTGG
GAGCAACTACCGGTTATTTAAAAGGAAACTCCGCTTCCTTCAACTTAGTTGGATTATTCGGAACAAAAACAAAA
TCTTCTGATTTTAATACAGCGAAGCTTGTTCCTAACATTGCTTTGAATCGAGCTGTGGTTGAGCTTTATACAGAC
ACTACCTTTGCTTGGAGCGTAGGTGCTCGTGCAGCTCTCTGGGAATGTGGGTGTGCAACGTTAGGA
