# Supplementary material for: Comparison of reverse hybridization and ompA sequencing methods applied on Chlamydia trachomatis strains from Tunisia
Source: Microbiologyopen. 2017 Dec 28;7(2):e00549. doi: 10.1002/mbo3.549 (PMC5911986; doi:10.1002/mbo3.549)
Supplement: Supplementary file 2 [file MBO3-7-na-s002.doc]

|  | **RHM** | | | **OSA** | | |
| --- | --- | --- | --- | --- | --- | --- |
|  | **CP** | **FSW** | **Total** | **CP** | **FSW** | **Total** |
| **Genovar** |  |  |  |  |  |  |
| **D** | 2 | 0 | 2 | 4 | 1 | 5 |
| **E** | 52 | 12 | 64 | 56 | 15 | 71 |
| **F** | 4 | 1 | 5 | 6 | 1 | 7 |
| **G** | 3 | 0 | 3 | 9 | 1 | 10 |
| **H** | 1 | 0 | 1 | 6 | 3 | 9 |
| **I** | 0 | 0 | 0 | 1 | 0 | 1 |
| **K** | 0 | 0 | 0 | 3 | 1 | 4 |
| **B+E** | 1 | 0 | 1 | - | - | - |
| **D+E** | 0 | 1 | 1 | - | - | - |
| **D+F** | 1 | 0 | 1 | - | - | - |
| **E+F** | 1 | 3 | 4 | - | - | - |
| **E+F+J+K** | 0 | 1 | 1 | - | - | - |
| **E+G** | 4 | 1 | 5 | - | - | - |
| **E+G+H+K** | 0 | 1 | 1 | - | - | - |
| **E+H** | 4 | 1 | 5 | - | - | - |
| **E+H+K** | 3 | 0 | 3 | - | - | - |
| **E+I** | 1 | 0 | 1 | - | - | - |
| **E+K** | 2 | 0 | 2 | - | - | - |
| **H+K** | 0 | 1 | 1 | - | - | - |
| **NI*** | 6 | 0 | 6 | - | - | - |
| **Total** | 85 | 22 | 107 | 85 | 22 | 107 |

**Table S1 :** Detailed distribution of the 107 *C. trachomatis* genovars for CP and FSW populations by both the Reverse Hybridization Method and the *ompA* sequencing.

* Not Identified genovars by RHM

- *ompA* sequencing enable to detect mixed infections
